# Who is WithMe? EEG features for attention in a visual task, with auditory and rhythmic support

**DOI:** 10.3389/fnins.2024.1434444

**Published:** 2025-01-10

**Authors:** Renata Turkeš, Steven Mortier, Jorg De Winne, Dick Botteldooren, Paul Devos, Steven Latré, Tim Verdonck

**Affiliations:** ^1^Internet Technology and Data Science Lab (IDLab), Department of Computer Science, University of Antwerp— Interuniversity Microelectronics Centre (imec), Antwerp, Belgium; ^2^Wireless, Acoustics, Environment & Expert Systems (WAVES), Department of Information Technology, Ghent University, Ghent, Belgium; ^3^Department of Art, Music and Theater Studies, Institute for Psychoacoustics and Electronic Music, Ghent University, Ghent, Belgium; ^4^Department of Mathematics, University of Antwerp—Interuniversity Microelectronics Centre (imec), Antwerp, Belgium

**Keywords:** EEG, visual attention, auditory support, rhythmic support, topological data analysis

## Abstract

**Introduction:**

The study of attention has been pivotal in advancing our comprehension of cognition. The goal of this study is to investigate which EEG data representations or features are most closely linked to attention, and to what extent they can handle the cross-subject variability.

**Methods:**

We explore the features obtained from the univariate time series from a single EEG channel, such as time domain features and recurrence plots, as well as representations obtained directly from the multivariate time series, such as global field power or functional brain networks. To address the cross-subject variability in EEG data, we also investigate persistent homology features that are robust to different types of noise. The performance of the different EEG representations is evaluated with the Support Vector Machine (SVM) accuracy on the WithMe data derived from a modified digit span experiment, and is benchmarked against baseline EEG-specific models, including a deep learning architecture known for effectively learning task-specific features.

**Results:**

The raw EEG time series outperform each of the considered data representations, but can fall short in comparison with the black-box deep learning approach that learns the best features.

**Discussion:**

The findings are limited to the WithMe experimental paradigm, highlighting the need for further studies on diverse tasks to provide a more comprehensive understanding of their utility in the analysis of EEG data.

## 1 Introduction

Understanding the human processing of multi-sensory stimuli in relation to attention has been of great interest in the last decades (De Winne et al., 2022). Indeed, detecting cognitive states and skills can help improve adaptive learning, in which the learning material and pace are adjusted to match some collected data about learners during a learning task (Mohamed et al., 2018). Moreover, identifying biomarkers that can be used to monitor attention, pleasure and reward, and understanding the relationship between these biomarkers and fine-tuning of stimuli (sound, image, rhythm) can enhance the interaction between humans and artificial intelligence (AI) agents, which is still lacking the degree of engagement and entrainment that characterizes interaction between humans. Such advances in human-centered AI approach open a wealth of applications in public security, health, revalidation, communication and information sharing, entertainment, etc. Some examples include driver fatigue detection (Wan et al., 2013), rhythmic auditory stimulation to help Parkinson's patients improve their gait characteristics and reduce the risk for falling (Moens et al., 2017), or music systems for synchronization (Moens et al., 2010) and gait retraining (to prevent running-related injuries; Van Dyck et al., 2015) which could be improved by selecting the best rhythmic or music stimulus at the right moment.

A promising methodology for the automated collection of data during a mental task includes the use of bio-sensors that could measure subjects' emotions, attention, and engagement in a non-invasive and non-intrusive way (Mohamed et al., 2018). In this work, we focus on capturing human attention from electroencephalography (EEG) bio-signals. EEG data measures oscillatory electrical brain activity at the macroscopic scale with high time resolution (Speckmann et al., 2011; Yu et al., 2016). EEG has been shown to have a strong potential to provide biomarkers for diagnoses in many neuropsychiatric disorders (da Silva, 2013; Yu et al., 2016), including attention deficit hyperactivity disorder (ADHD; Lubar, 1991; Loo and Barkley, 2005; Liu et al., 2015; Janssen et al., 2017; Kiiski et al., 2020), but also as indicators of attention during different visual and cognitive tasks (Mulholland, 1969; Ray and Cole, 1985; Harmony et al., 1996; Klimesch et al., 1998; Sauseng et al., 2005; Busch and VanRullen, 2010; Liu et al., 2013; Abiri et al., 2019; Jin et al., 2019).

It is commonly understood that a crucial step in EEG processing is to extract relevant features for the considered application (Xu et al., 2021). Moreover, the study of EEG data, similarly to other neural data, is further complicated by the high degree of cross-subject variability (due to differences in how the information is represented in the brain, e.g., in terms of the representation of stimulus and activity in the brain), and presence of noise (due to changes in machine calibration, spurious participant movements, and environmental conditions) (Rieck et al., 2020).

The goal of this paper is to investigate which type of representations or features of EEG data are the most associated with human attention. We will consider a number of different representations of EEG data (Section 2.2, Supplementary Material Section 2), including both the features obtained from the univariate time series from a single EEG channel (and then concatenated across channels), such as time domain features and recurrence plots, as well as representations obtained directly from the multivariate time series, such as global field power or functional brain networks. These two groups of methods are related to the two key principles that help in understanding brain-behavior relationships: segregation, which assumes that the cerebral cortex can be divided into distinct modules, each with its unique structure and functionality; and integration, which assumes that no brain region functions in isolation but rather requires interactions and information exchange between different regions (Mohamed et al., 2018). Since we aim to deal with the issue of person-to-person variability in the EEG data, we found it particularly interesting to consider some topology-based features. Indeed, persistent homology (PH), the main tool of topological data analysis (TDA, Supplementary Material Section 1) can be made invariant under different type of transformations (such as translation, rescaling, stretching, or even non-affine deformations), and the stability theorems (Cohen-Steiner et al., 2007) imply that the same is true for robustness to noise. Persistent homology has been widely applied in neuroscience: we provide a review of relevant literature in the background on TDA (Supplementary Material Section 1), and in the description of the PH-based pipelines on univariate (Section 2.2.4) and multivariate (Section 2.2.8) time series.

We compare the performance of the different EEG representations on the WithMe data (Section 2.1), obtained from a modified digit span experiment. The performance is evaluated as the Support Vector Machine (SVM) accuracy on the features (Section 2.4). As a baseline, we also include benchmark EEG-specific models which are shown to work well for the WithMe data (Mortier et al., 2023), including a deep learning architecture that learns the best features for the task at hand from the EEG multivariate time series. In order to investigate the cross-subject variability, we consider three different scenarios: the accuracy is evaluated on the model trained on the same participant, on seen or on new participants. The results are summarized in Section 3, and in Section 4 we position them relative to the literature, and discuss the main take-aways and resulting directions for future work.

## 2 Materials and methods

### 2.1 WithMe EEG data acquisition

**Figure 1:**
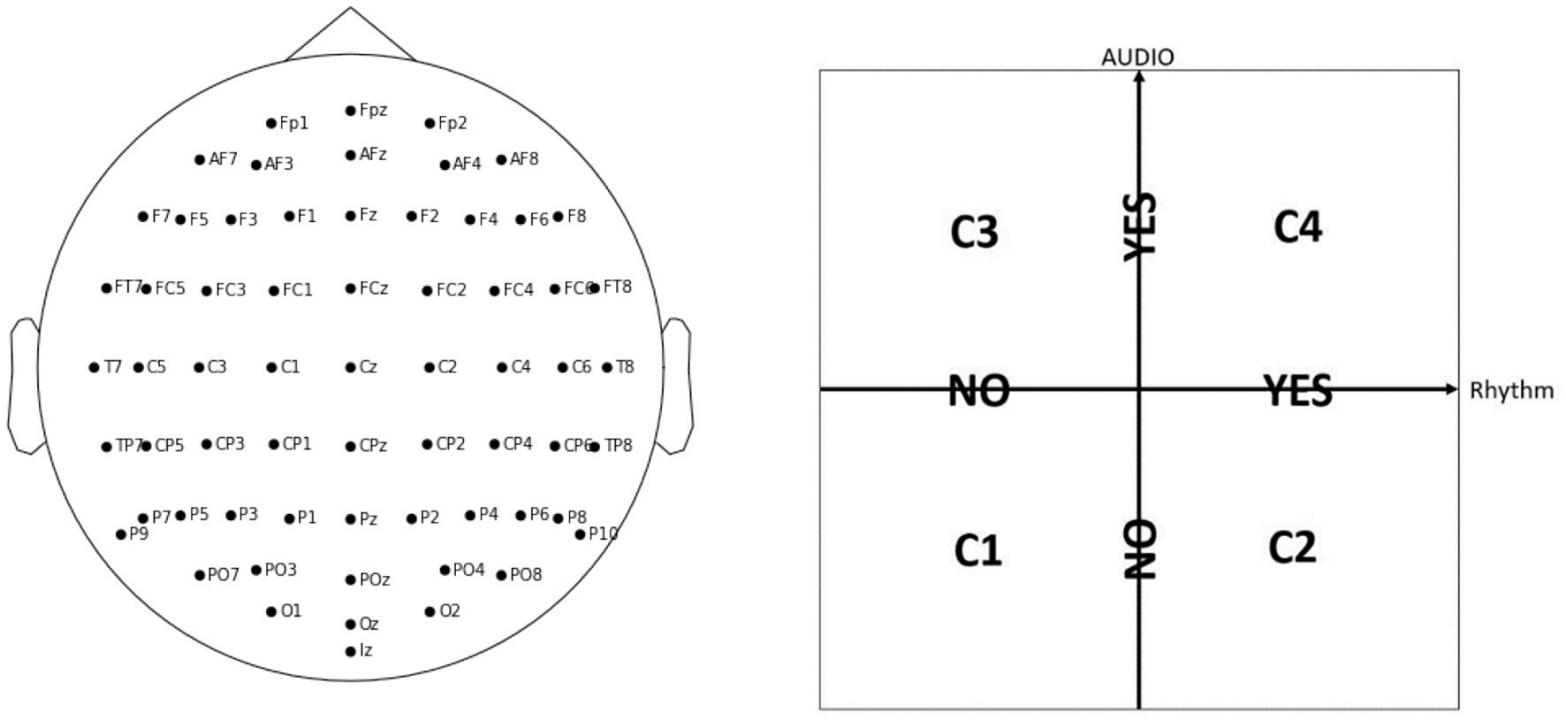
In the WithMe experiment, 64 EEG electrodes are considered, according to the 10–10 system **(left panel)**. Every sequence of numbers is shown to each participant under four different conditions, C1, C2, C3, or C4, which indicate the presence of auditory and/or rhythmic support **(right panel)**.

The experiment includes 42 participants EP01-EP42 (20 female and 21 male, with mean age of 23.71 ± 2.69 years, with no visual or hearing difficulties), who have 64 electrodes positioned on their scalp according to the EEG 10 − 10 system (Figure 1, left panel). The EEG data was acquired with the BioSemi ActiveTwo^1^ amplifier, together with their caps and gel-based electrodes, and sampled at 2,048 Hz. Each participant is shown 30 sequences of 10 stimuli on a computer screen: 5 Targets (black digit in a circle), and 5 Distractors (dark gray digit in a circle, or an empty circle), with each digit presented with equal probability (for an example sequence, see Table 1). Each of the sequences is shown under four different conditions C1, C2, C3, or C4, in a pseudo-randomized manner, that differ with respect to presence of audio and/or rhythm with no special mention about them made to the participants, in order not to draw their attention to it (Figure 1, right panel). We note that, for conditions C2 and C4 where there is rhythm, each participant is first shown 5 induction stimuli in each sequence to induce the rhythm, but these are ignored in our analysis. Therefore, every participant sees in total 30 × 10 × 4 = 1200 Target or Distractor stimuli. Every stimulus is visible for 200 ms, and the inter-stimulus interval is on average 1.25 s. The task is to remember the Targets, and the participants need to give their oral responses after the complete sequence is presented. The participants do not know that there are always 5 Targets: they are told they will see “5-7 black numbers,” in order to not be able to predict the end of the Target stimuli, but to rather keep focusing until the end of the sequence.

**Table 1:** An example of a sequence of stimuli shown to each experimental participant on a computer screen, with the digits appearing one by one, Targets in black and Distractors in gray.

**Sequence**	**⑧** ⑥ ⑥ ⑥ ⑨ ○ ⑨ ⑦ ⑤ **④**
Targets	86974
Distractors	66 95
Answer	86694

For the given answer, the participant obtains the following scores on the 10 shown Target and Distractor stimuli: score(*T*_1_) = 0, score(*T*_2_) = 0, score(*T*_3_) = 0.2, score(*T*_4_) = 1, score(*T*_5_) = 0, score(*D*_1_) = -1, score(*D*_2_) = -1, score(*D*_3_) = -1, score(*D*_4_) = 0, score(*D*_5_) = 0.

The WithMe experiment is a novel working memory paradigm that is somewhat inspired by the digit span, oddball and pip-and-pop tasks. The magical number 7 ± 2 is the average digit span of healthy adults (Miller, 1956), so that a sequence of five digits should be fairly easy to remember for a young adult. The digit span memory task is modified by including Distractors between Target stimuli. In the oddball attention task, a series of repetitive standard stimuli are infrequently interrupted with a rare oddball stimulus that the participant is instructed to focus on. In the WithMe experiment, however, there is an equal number of Target and Distractor stimuli, so there are no oddballs. The pip-and-pop attention paradigm makes the Targets immediately noticeable among the surrounding items (they “pop out”), but the difference between the WithMe Target and Distractor stimuli is very subtle. In summary, the WithMe experimental design complicates the three paradigms, in order to allow to more easily observe the effect of added support on attention. Further details can be found in the first paper that introduces the WithMe experiment, and provides an in-depth analysis of behavioral factors (such as musical education, audiovisual dominance, noise sensitivity, gender, age, etc.) on the influence of audio and/or rhythm on attention for the WithMe data (De Winne et al., 2022).

The data is pre-processed according to standard techniques: the amplitude values are referenced to the average of the both earlobes, bad channels are detected after visual inspection and then interpolated using the spherical spline method (Perrin et al., 1989),^2^ notch filter is applied at 50 Hz, and bandpass filter between 0.2 and 100 Hz. The data is then epoched from -0.2 s to 1 s, and a visual inspection of ICA components is performed to remove artifacts. Finally, we downsample the time series with the subsampling period of 50, resulting in 60 time steps, so that each time step corresponds to 1,200 ms/59 = 20.34 ms. In most situations, downsampling the results to 40 or 50 Hz (thus, one time point every 25 or 20 ms) maintains the advantages of downsampling with minimal loss of information (Cohen, 2014). Finally, the EEG amplitude values are cut off within range [−50 μ*V*, 50 μ*V*]. The WithMe dataset can then be seen as a 42 × 1200 × 64 × 60 matrix:

- 60 time steps (within 1.2*s*),
- 64 EEG channels,
- 1, 200 epochs, i.e., EEG multivariate time series reflecting a single stimulus, across channels,
- 42 participants.

Some examples of the WithMe EEG multivariate time series across 64 electrodes, for a single participant and a single stimulus, are visualized in Figures 5, 8.

### 2.2 Multivariate time series analysis

**Figure 2:**
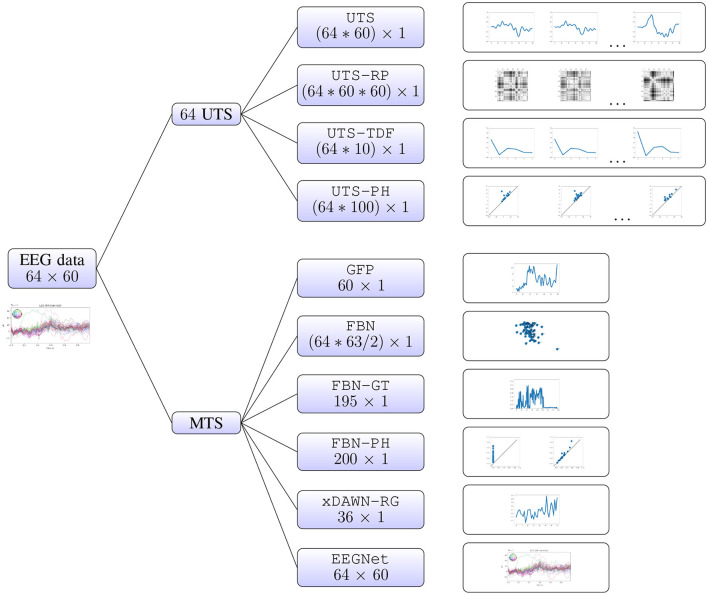
Multivariate time series analysis. A WithMe data observation, an EEG multivariate time series across 64 electrodes and 60 time steps (reflecting the brain signals for some participant and stimulus shown on the screen) can be represented with different types of features. We can consider the 64 univariate time series (UTS) for each EEG electrode, extract features from each of them separately, and concatenate the information **(top branches)**. It is also possible to calculate the features from the multivariate time series (MTS) itself, that rely on the relationship between the 64 EEG channels **(bottom branches)**.

In this section, we describe in detail the different approaches to multivariate time series analysis that we will evaluate in the computational experiments. These methods rely on the different types of features, or representations of multivariate time series, that belong to two different groups. Firstly, one can consider the individual univariate time series (for each EEG electrode), and concatenate the information extracted from each of them separately (Sections 2.2.1–2.2.4). Alternatively, we can focus on the relationship between the univariate time series (i.e., relationship between different brain regions), which we summarize in a few different ways (Sections 2.2.5–2.2.10). The latter approaches include the baseline xDAWN-RG model (Congedo et al., 2017), the IEEE Neural Engineering Conference 2015 Brain Computer Interface (BCI) challenge winner, and EEGNet (Lawhern et al., 2018), a benchmark deep learning architecture for EEG signal processing and classification which learns the best representation for the given task, that have both been shown to perform well on the WithMe data (Mortier et al., 2023). A visual summary of all of the different approaches is given in Figure 2.^3^

#### 2.2.1 Univariate time series (UTS)

Probably the most straightforward way to transform a multivariate time series into a vector is to concatenate the univariate time series across all features. For instance, a WithMe multivariate time series across 64 electrodes and 60 time steps (matrix of shape 64 × 60) can be represented as a vector of length 3840 = 64*60.

#### 2.2.2 Recurrence plots of univariate time series (UTS-RP)

A recurrence is a time the trajectory returns to a location it has visited before. For a univariate time series, the recurrence plot is a matrix of distances in the signal between every pair of points in time. A WithMe multivariate time series across 64 electrodes and 60 time steps (matrix of shape 64 × 60) can in this way be represented with a vector of length 230400 = 64*60*60, corresponding to the flattened 60 × 60 recurrence plots, concatenated across 64 EEG electrodes. Recurrence plots have been employed in EEG analysis, for example for emotion recognition (Bahari and Janghorbani, 2013), or to differentiate between seizure-free, pre-seizure and seizure states in genetic absence epilepsy rats (Ouyang et al., 2008).

#### 2.2.3 Time-domain features of univariate time series (UTS-TDF)

Instead of looking at the whole univariate time series for each channel (UTS pipeline, Section 2.2.1), we can also only extract some statistics about the time series, such as their maximum, minimum, mean and variance. Next to considering the largest peaks in the time series, we include the peaks from within certain intervals, as these are related to the so-called event-related potentials (ERPs). An ERP is a stereotyped brain response to a specific sensory, cognitive, or motor stimulus. ERP waveforms consist of a series of positive and negative voltage deflections, which are related to a set of underlying components. Most components are referred to by a letter (N/P) indicating polarity (negative/positive), followed by a number indicating either the latency in milliseconds or the component's ordinal position in the waveform:

- N100 or N1: This is the first substantial peak in the univariate time series. It is a negative-going peak typically occurring about 100 ms after a stimulus is presented, but may exhibit a peak anywhere between 80 and 120 ms (280 and 320 ms after the start of our time series, see Section 2.1). We therefore calculate the minimum of the waveform within this range (between time steps *t* = 13 and *t* = 16 in our time series).
- P100 or P1: This is a positive extreme occurring about 100 ms after a stimulus is presented, but may exhibit a peak anywhere between 80 and 120 ms. We therefore calculate the maximum of the time series within this range (between time steps *t* = 13 and *t* = 16 in our time series).
- P200 or P2: This is the second substantial peak in the time series, which often occurs about 200 ms after the stimulus onset. We calculate it as the maximum of the waveform between 150 and 275 ms, i.e., between time steps *t* = 17 and *t* = 24 in our time series.
- P300 or P3: This is the third substantial positive-going peak in the waveform, occurring about 300 ms after a stimulus is presented. We calculate it as the maximum of the waveform between 250 and 500 ms, i.e., between time steps *t* = 22 and *t* = 35 in our time series.

A review of EEG/ERP applications can be found in Nidal and Malik (2014). In particular, multiple ERPs were found to be associated with mind-wandering (Jin et al., 2019), or different stages of attention (Abiri et al., 2019). Note that the EEG reflects thousands of simultaneously ongoing brain processes, making it challenging to see the brain response to the event of interest in the EEG recording of a single trial (Blankertz et al., 2011). To see the brain's response to a stimulus, the experimenter commonly conducts many trials and averages the results, causing random brain activity to be averaged out and the relevant waveform to remain (Cohen, 2014). However, in this work we focus on applications that aim to predict the attention from the given EEG time series corresponding to some stimulus, and we therefore extract the given metrics for each EEG epoch. We represent each of the univariate time series with 10 time-domain features: maximum, minimum, mean, variance, skewness, kurtosis (similar to Mohamed et al., 2018) N1, P1, P2, and P3 peaks, and then concatenate the information across EEG electrodes. In this way, a WithMe 64 × 60 multivariate time series is represented with a vector of length 640 = 64 * 10.

#### 2.2.4 Persistent homology of univariate time series (UTS-PH)

The shape of the EEG wave has been shown to contain useful information about the state of the brain (Kannathal et al., 2005). For this reason, we represent EEG time series for each channel with its persistent homology with respect to the lower-star filtration (Supplementary Material Section 1) directly on the signal, i.e., on the function *f*:{*t*_1_, …, *t*_*n*_} → ℝ. Persistent homology with respect to such a filtration measures the relative height of the peaks of the EEG signal, but not their width, so that it is invariant to expansion and contraction in the time axis direction (Supplementary Material Section 1, Figure S2; Supplementary Material Section 1, Figure S4, row 5, columns 2 and 4). Moreover, addition of noise to the signal results in minor changes of PH (Supplementary Material Section 2, Figure S4, row 5, columns 2 and 5). This makes PH an interesting candidate for overcoming individual differences across subjects (Dindin et al., 2020).

A similar pipeline is employed for the epileptic seizure, autism and arrhythmia detection from EEG or ECG in Wang et al. (2015), Wang et al. (2019), Majumder et al. (2020), and Dindin et al. (2020). In this work, we represent the WithMe EEG univariate time series with their 0-dimensional 10 × 10 persistence images (Supplementary Material Section 1), so that an EEG epoch for 64 channels and 60 time steps results in a vector of length 6, 400 = 64 × 10 × 10.

#### 2.2.5 Global field power (GFP)

The representations above (Sections 2.2.1–2.2.4) focus on the features from the univariate time series. However, the interactions between the different time series (i.e., different brain regions reflected by the EEG channels) might contain (more) meaningful information. One of the simplest ways to summarize this relationship between the time series is via the global field power (GFP). GFP is a measure of the scalp field strength and corresponds to the standard deviation of the signal across electrodes at each time point (Skrandies, 1990). Thus, it is a one-dimensional time series capturing the spatial variability of the signal across sensor locations. The WithMe 64 × 60 multivariate time series is thus represented as a vector of length 60. GFP has found applications in studies of perceptual, attentional, cognitive and drug-related aspects of information processing (Michel et al., 1993).

#### 2.2.6 Functional brain network (FBN)

From a multivariate time series, one can construct a graph or network, with vertices or nodes which reflect the different univariate time series, and the edges which describe some relationship between them. In the context of (WithMe) EEG multivariate time series, the nodes correspond to the different EEG electrodes or brain regions, and the weights of the edges correspond to some measure of connectivity between them, with the resulting graph commonly referred to as a functional brain network (Stolz et al., 2018). These functional brain networks provide a new understanding of the characteristics of the brain, since different cognitive or perceptual tasks require a coordinated flow of information within networks of functionally specialized brain areas (Bastos and Schoffelen, 2016).

Indeed, changes in the topology of EEG functional brain networks appear to accompany a series of neurological and psychiatric disorders, such as stroke damage (de Vico Fallani et al., 2009), schizophrenia (Jalili and Knyazeva, 2011b; Micheloyannis et al., 2006), amyotrophic lateral sclerosis (ALS; Fraschini et al., 2016) or Alzheimer's (Stam et al., 2007; Jalili, 2016; Yu et al., 2016), and can therefore be used as diagnostic markers for these conditions (Stolz, 2014). Moreover, several studies have suggested that EEG interregional correlations are associated with conscious cognitive processing and active perception (de Vico Fallani et al., 2009; Tóth et al., 2019).

The correlation between time series (i.e., connectivity between brain regions) can be calculated in many different ways, such as cross-correlation, coherence, and synchronization likelihood. In our computational experiment, we will consider the common Pearson product-moment correlation coefficients (Jalili and Knyazeva, 2011a). The distance *d*_*ij*_ between the two univariate time series *i* and *j* is then calculated as *d*_*ij*_ = 1−*p*_*ij*_, where *p*_*ij*_ is the Pearson correlation. As input for this FBN pipeline, we will consider the distance matrix itself, above the diagonal and flattened into a vector, similarly to Rathore et al. (2019). The WithMe 64 × 60 multivariate time series thus results in a 64 × 64 distance matrix, that is then flattened into a vector of length 2, 016 = 63*64/2.

#### 2.2.7 Graph theory of functional brain networks (FBN-GT)

Instead of feeding the complete correlation or distance matrix to a machine learning algorithm (FBN pipeline, Section 2.2.6), it is common to analyze such a matrix (or functional brain network in the neuroscience context) using graph theory. Typically, the weighted graph, i.e., the correlation or distance matrix is thresholded at a prespecified level to produce the binary adjacency matrix that only indicates if a connection between vertices exists (Lee et al., 2011). Then, the corresponding graph topology of the binary matrix can be characterized by calculating the graph metrics of interest that characterize the functional integration and segregation.

Indeed, significant differences across some graph theory metrics have been found between EEG brain networks for control subjects and patients of a range of neurological and psychiatric disorders (Bullmore and Sporns, 2009; Fraschini et al., 2016) (such as stroke, multiple sclerosis, Parkinson's, epilepsy or depression). Moreover, in the healthy brain, individual variability in cognitive functions, learning a new task, or the predisposition to learn have been correlated with specific patterns of network connectivity (Khalid et al., 2014). In our computational experiments, we consider the commonly used assortativity degree, average path length, edge connectivity, and for each vertex, its degree, betweenness, and eccentricity,^4^ that provide insights into how different brain regions communicate and interact:

- Assortativity measures the tendency of vertices to connect with other vertices with a similar degree (number of adjacent edges, see below). High assortativity means that highly connected vertices (hubs) tend to be connected with other hubs. In the brain, this can indicate a resilient and efficient network because hub-to-hub connectivity is critical for global communication. A low assortativity may imply a more vulnerable network, where the failure of a single hub can more easily disrupt information flow.
- Average path length is self-explanatory, it is the average shortest path length between all vertex pairs. Shorter path lengths indicate more efficient global communication across the network. In the brain, a network with a shorter average path length facilitates fast information flow across distant brain regions, which is critical for efficient integration of information between different functional systems.
- Edge connectivity between two vertices is the number of edges that have to be removed in order to disconnect the two vertices into two separate components, or equivalently, to eliminate all paths between them. The edge connectivity of a graph (or group adhesion) is the minimum of the edge connectivity of every pair of vertices in the graph. Multiple connections between regions create redundant routes, which are important for ensuring reliable communication and cognitive flexibility. High edge connectivity thus implies that brain networks can resist damage, maintaining communication pathways and ensuring stable function.
- The degree of a vertex is its most basic structural property, the number of edges that are connected to that vertex, measuring how many direct connections a vertex has with other vertices in the graph. Vertices with a high degree are considered hubs and may play a key role in information integration and transmission across the brain. In functional brain networks, a region with a high degree is likely involved in widespread functional connectivity and might be important for global network communication.
- The betweenness centrality of a vertex is the number of shortest paths going through it. Vertices with high betweenness centrality are key to information flow across the network because they act as critical “bridges” between different brain regions or modules. Such verticess may be vulnerable to damage, as their failure could disrupt communication between distant parts of the brain.
- Eccentricity of a vertex is the shortest path distance from the farthest other vertex in the graph. Eccentricity provides information about the relative position of a brain region in the broader network and its role in global communication. High-eccentricity vertices are more isolated or peripheral, often supporting localized, specialized processing within functional modules, while low-eccentricity vertices integrate information from various parts of the brain.

To obtain the graph adjacency matrix, the normalized distance matrix (see FBN pipeline in Section 2.2.6) is thresholded at 0.1.^5^ For a WithMe EEG multivariate time series across 64 channels, i.e., a 64 × 64 functional brain network (distance matrix), this results in a vector of length 195 = 1+1+1+64*(1+1+1).

#### 2.2.8 Persistent homology of the functional brain network (FBN-PH)

Studying the correlations using graph theory or network science (FBN-GT pipeline, Section 2.2.7) suffers from methodological problems. Firstly, finding a proper threshold is one of the crucial issues, since the graph structure drastically changes depending on how to threshold a connectivity matrix. For example, most graph characteristics depend on the number of edges in the graph, and the estimated graph topology is therefore biased by the choice of the threshold. This hampers a meaningful comparison of graph topology between individuals or groups. Some of the proposed thresholding methods, such as the multiple comparisons correction and the sparsity control, assume that the strongly connected edges are only important; however, it is suggested that the weakly connected edges may also have discriminative information between networks (Bassett et al., 2012; Lee et al., 2012). The choice of threshold has a major influence on the resulting graph (Gracia-Tabuenca et al., 2020) and inevitably leads to a loss of information. Determining the threshold can be based on the statistical significance by the false discovery rate or by fixing the graph metrics such as number of vertices and edges. However, these methods are fairly *ad-hoc* and everyone seem to use different thresholding techniques. This arbitrariness is demonstrated in Lee et al. (2011, Figure 1), where it is shown that the number of edges, the number of connected components and smallworldness of a brain network substantially change depending on the threshold: by varying the threshold, the topology changed to random-like, small-world and clustered network. In addition, it was shown that the clustering coefficient, modularity, efficiency, efficiency-cost show and assortativity of a brain network change greatly across different thresholds (reflected by the network cost, i.e., the total number of edges; Joudaki et al., 2012, Figures 3–7), and the same was shown to be true for efficiency, clustering coefficient, small-worldness, modularity, vertex and edge betweenness centrality, variance of vertex degrees, assortativity, and synchronizability in Jalili and Knyazeva (2011a, Figures 5–8). Ideally, graphs should be characterized across a broad range of thresholds (Rubinov and Sporns, 2010).

Moreover, in many real systems, dyadic relationships between pairs of vertices fail to accurately capture the rich nature of the system's organization, e.g., cognitive functions appear to be performed by a distributed set of brain regions and their interactions (Giusti et al., 2016). Furthermore, another drawback of the common graph theory approaches is that they require 2-dimensional embedding of structures that might otherwise be of higher dimension (Bendich et al., 2016).

Persistent homology (PH) of a graph goes beyond graph-theoretic analysis by describing the architecture of a graph in more flexible ways, that investigates the persistence of relationships between graph vertices across multiple scales (Anderson et al., 2018). Instead of trying to determine one fixed optimal threshold, PH allows us to look at the topological changes of graphs while increasing the threshold continuously. Persistent homology represents the weighted graph with a finite number of nested binary graphs over every possible threshold. In contrast to standard methods of graph or network analysis, PH also encodes higher order connections and thus allows to go beyond pairwise connections; this is helpful for gaining global understanding of low-dimensional structures in graphs (Stolz et al., 2018). Indeed, experimental results in Guo et al. (2022) show that compared with the existing methods, PH can extract the topological features of brain networks more accurately and improves the accuracy of diagnostic and classification.

The first papers that deal with PH of brain networks (Lee et al., 2011, 2012) demonstrate differences between the local connectivity structures in functional brain networks for attention deficit hyperactivity disorder (ADHD) and autism spectrum disorder (ASD), and later, in a depressed brain (Khalid et al., 2014; Yoo et al., 2014). Furthermore, PH metrics have been employed to investigate how the topological architecture of brain networks is related to cognitive function, behavior and personality (Anderson et al., 2018; Liu et al., 2021; Yoo et al., 2016). In this paper, we employ PH with respect to the rank filtration (Section A.1) on the functional brain networks. This is useful in neuroscience applications, where correlations cannot be assumed to give a precise definition of the distances between graph nodes, and the PH defined in such a way remains unchanged under nonlinear monotonic transformations of the distances (see Supplementary Material Section 1, Figure S3). More precisely, we concatenate both the 0- and 1-dimensional 10 × 10 persistence images (that respectively reflect the connected components or clusters, and loops) for each graph, so that a WithMe 64 × 64 functional brain network is represented with a vector of length 200 = 10*10+10*10.

#### 2.2.9 xDAWN-RG (xDAWN-RG)

As one of the baselines, we consider xDAWN-RG, one of the benchmark techniques for classification of multivariate bio-signals, like EEG, MEG, or EMG. xDAWN-RG was the winner of the IEEE Neural Engineering Conference Brain Computer Interface (BCI) challenge (Mattout et al., 2014), whose goal was to detect errors during a spelling task, given subject's EEG data. It consists of applying xDAWN spatial filters (Rivet et al., 2009), calculating covariance matrix between the EEG channels to encode their statistical dependencies (Barachant and Congedo, 2014; Congedo et al., 2013), selecting the channels via Riemannian Geometry (RG) (Barachant and Bonnet, 2011), and projecting the reduced covariance matrices in the tangent space (Barachant et al., 2011, 2013). A WithMe 64 × 60 multivariate time series is transformed into a 8 × 8 covariance matrix, which is then projected into a vector of length 36 = (8+1)*8/2.

#### 2.2.10 EEGNet (EEGNet)

As the main baseline, we consider EEGNet (Lawhern et al., 2018), a benchmark deep learning architecture for EEG signal processing and classification, with default parameter values. EEGNet is a convolutional neural network (CNN) that learns the best representation for the given task directly from the multivariate time series, with each data observation corresponding to the matrix of univariate time series across channels. Therefore, the raw WithMe 64 × 60 multivariate time series is fed directly to the model.

### 2.3 Attention score

The focus of this paper and the WithMe data acquisition (Section 2.1) is attention recognition. Remember that each experimental participant is shown 30 sequences of 10 Target or Distractor numbers on the screen, and is instructed to list the Targets in the correct order of appearance. We are thus interested to what degree the different pipelines (Section 2.2) can predict how well a participant was able to remember the Targets and ignore the Distractors from the EEG data. To do so, we define a simple scoring function, which reflects the digit recall accuracy, i.e., how well a participant was paying attention to the given stimulus.

Note firstly that the attention performance is not always conclusive, since the Targets and Distractors in a sequence are not necessarily unique: e.g., if a sequence contains digit 5 both as a Target and as a Distractor, and a participant reports 5, we do not know if the participant correctly remembered the target, or did not properly ignore the distractor. We assign such stimuli a value of -1.

We define the scoring function for the remaining, well-behaved stimuli to take values in [0, 1]. The score of 0 indicates perfect performance: Target stimulus is given in the participant's answer in the correct position, or the Distractor stimulus was properly ignored. On the other extreme, the score of 1 means that the participant did not remember the Target, or has listed the Distractor in their answer. The scores of 0.2, 0.4, 0.6, or 0.8, that are only possible for the Targets, aim to capture that a participant remembered the Target, but at the wrong position; the value of the score indicates how wrong is the provided answer. Note, however, that e.g., a score of 0.2 for some Target stimulus does not necessarily imply that the subject was not paying “perfect” attention, since it might rather be that they were not attentive during a previous Target that they thus did not include in their answer. Since a stimulus might appear multiple times in a sequence, note also that the scoring function does not necessarily always correctly reflect the attention, and a participant might simply “get lucky” and report the number correctly.

Therefore, our simple scoring function is defined as follows:


(1) $$\begin{array}{l} \text{score}\left(T\right)=\left\{ \begin{array}{ll} 0 & T\in \text{answer, at the right position}\\ 0.2 & T\in \text{answer, wrong by one position}\\ 0.4 & T\in \text{answer, wrong by two positions}\\ 0.6 & T\in \text{answer, wrong by three positions}\\ 0.8 & T\in \text{answer, wrong by four positions}\\ 1 & T\notin \text{answer}\\ -1 & T\text{ appears in answer less than in }\text{Targets} \end{array}\right. \end{array}$$

(2) $$\begin{array}{l} \text{score}(D)= \end{array}\left\{ \begin{array}{l} 0\;D\text{ appears in answer less than or same as}\\ \text{ in }\text{Targets}\\ 1\;D\text{ appears in answer more than or same in}\\ \;\text{Targets}+\text{Distractors}\\ -1\;D\text{ appears in answer less than in}\\ \;\text{Targets}+\text{Distractors},\text{ or }D\text{ is empty} \end{array}\right.$$

**Figure 3:**
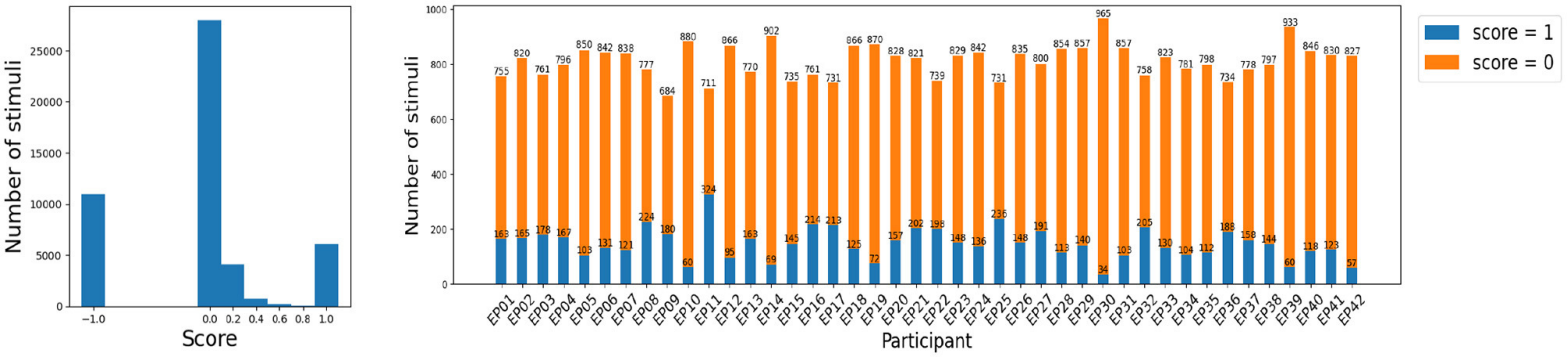
Distribution of scores. The number of inconclusive stimuli with score = -1 is not negligible **(left plot)**, but removing them does not lead to a major data loss, in particular since it allows to focus on the EEG epochs with a meaningful attention score. The number of EEG epochs with score in {0.2, 0.4, 0.6, 0.8} is too small for a score prediction task **(left plot)**, so that this data is also removed. In this way, score prediction amounts to classification between score = 0 (attentive) and score = 1 (inattentive) stimuli or EEG epochs.

An example of a sequence, a participant's answer and their score for each stimulus is given in Table 1. Figure 3 shows that the large majority of the stimuli yield the perfect score = 0: the Target is remembered at the correct position, or the Distractor is appropriately ignored. This is as expected since, as we discussed earlier, the literature suggests that a sequence of five digits should be fairly easy to remember for young adults.

### 2.4 Experimental set-up

The classification is done with a linear SVM on the features obtained in the pipelines, except for EEGNet that classifies the multivariate time series directly. According to a recent review (Lotte et al., 2018), due to its good performance, SVM is among the most popular types of classification algorithms for EEG. Moreover, we also want to evaluate to what extent are the different pipelines able to deal with the person-to-person variability, as this is an important challenge of EEG data (Section 1). To this end, we consider three different experimental scenarios, that differ in the train and test data used for classification:

- Classification per participant: We start with the simplest scenario, when the model is trained on 70% of data (randomly chosen EEG epochs) for a single participant, and the test data corresponds to the remaining 30% of multivariate time series for that same participant.
- Classification on seen participants: Next, we train the models on 70% of randomly chosen multivariate time series, and test on the remaining data. In this case, the train and test data consists of all (and therefore, the same) participants.
- Classification on new participants: Finally, we train the models on all the EEG data from 70% of the experimental participants, and test on the complete data for the remaining 30% participants. Here, the test data consists of new participants compared to the train data.

For each of the three scenarios, we consider three different splits between train and test data, which are the same across different pipelines. The drop in accuracy from the test data consisting of seen and new participants (the last two scenarios above) can give an idea of how well a pipeline is able to avoid the issue of cross-subject variability. Note that the size of data differs greatly between the first, and second and third experimental scenario, since the former is limited to the EEG data from a single participant. The data size for each experiment is explicitly mentioned in Section 3.

Finally, note that the UTS-RP, UTS-TDF and UTS-PH pipelines that extract features from the univariate time series yield a large number of features (relative to the number of data observations, e.g., 230400 UTS-RP features for 1200 data samples in the first experimental scenario where we focus on an individual subject). To reduce the computational efforts, we limit these pipelines to the features from the 10 most important EEG channels, identified by the UTS pipeline: we take these to be the EEG channels with the largest values of the linear SVM coefficients trained on the complete data. Removal of noisy of irrelevant channels can also make the model less prone to overfitting (Montoya-Mart́ınez et al., 2019), but we did not observe important differences when using only a subset of features.

For some of the classification problems, the data is very unbalanced: for instance, the number of EEG epochs with score=1 is quite small in comparison with score = 0 (Figure 3), so that even a random guess yields a very high accuracy. For this reason, we consider a balanced accuracy, which is calculated as the average between the true positive rate or recall $\frac{TP}{TP+FN}$, and the true negative rate $\frac{TN}{TN+FP},$ where *TP, TN, FP* and *FN* are respectively the number of true positives, true negatives, false positives and false negatives. If the data is well-balanced, the accuracy and balanced accuracy tend to converge to the same value. We opt for an adjusted classification accuracy in order to allow for a fair comparison across participants (which would not be the case if we oversampled some epochs for some participants), simultaneously avoiding data loss due to undersampling.

## 3 Results

### 3.1 Score prediction

The goal of this subsection is to evaluate to what extent can EEG signals predict the attention score (Section 2.3) that reflects how well a person has remembered the given Target, or ignored the given Distractor stimulus, shown on the computer screen. We thus perform the classification separately for Target and Distractor stimuli, as the score might reflect different types of attention. We limit the data to EEG multivariate series with score = 0 (attentive) and score = 1 (inattentive), in order to focus only on the stimuli for which we are fairly confident whether a participant was paying attention. Indeed, as we discuss in Section 2.3, the data samples with score=-1 are inconclusive, and score ∈{0.2, 0.4, 0.6, or 0.8} might often be misleading. Moreover, there is only a limited number of stimuli with a score of 0.2, 0.4, 0.6, or 0.8 (Figure 3, left panel) so that any classifier would struggle to learn to recognize such signals. This results in a somewhat different number of appropriate EEG epochs across participants (Figure 3, right panel). To make the comparisons across participants fair, we select the same number of Target and Distractor EEG epochs for each participant. Participant EP09 has the minimum number of Target epochs with a conclusive score (303), whereas participant EP17 has the least conclusive Distractor epochs (357). We therefore randomly choose 303 Target and 357 Distractor epochs for every participant. Note that, since we are evaluating the balanced classification accuracy (Section 2.4), we do not further enforce the same number of Target and Distractor epochs, in order to avoid further data loss.

**Figure 4:**
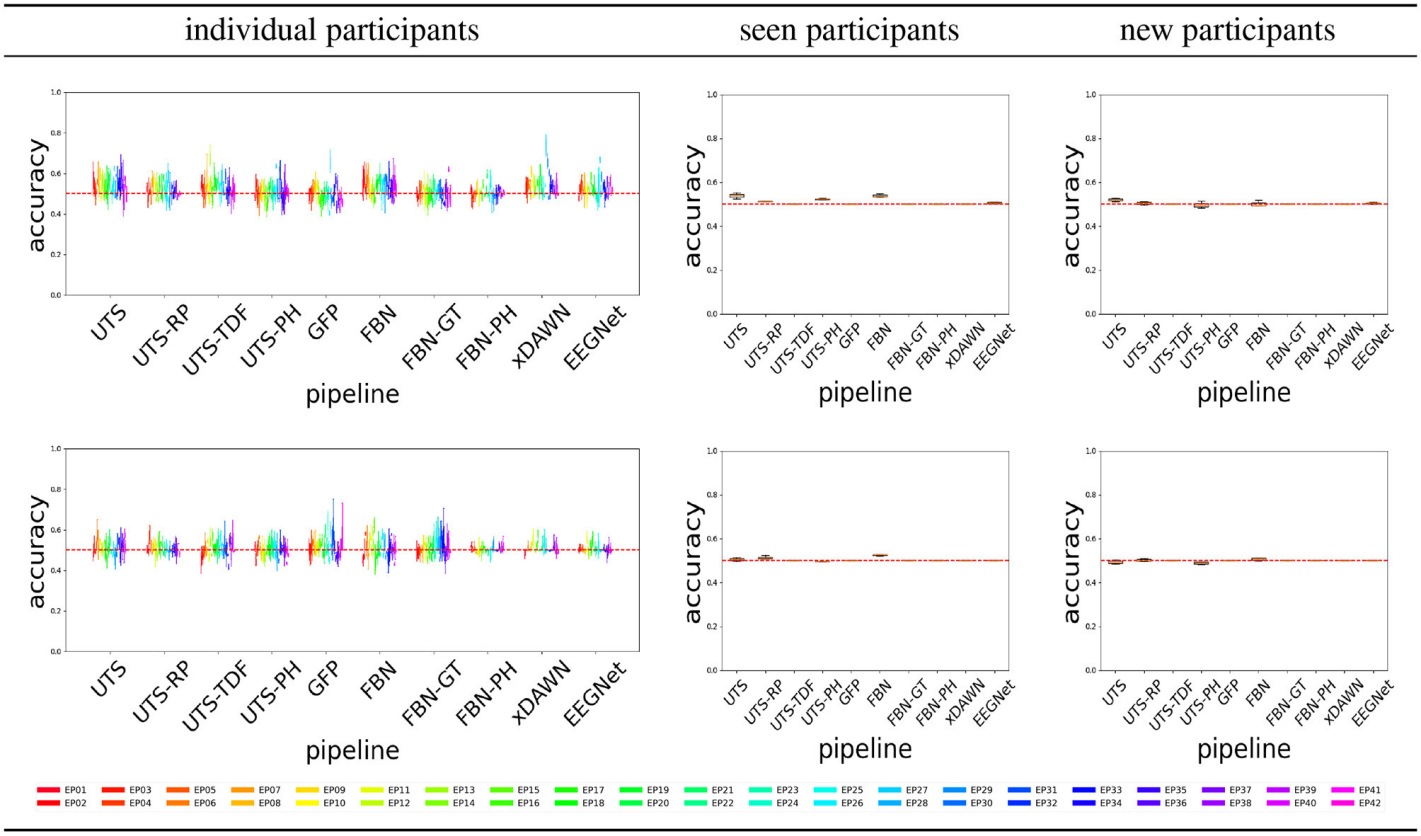
Score 0 (attentive) vs. 1 (inattentive) classification accuracy across the pipelines, for the Target **(top row)** and Distractor **(bottom row)** stimuli.

Figure 4 shows the balanced accuracy for score = 0 vs. score = 1 classification for the different pipelines (Section 2.2), for the three different experimental scenarios, i.e., train and test data (Section 2.4). In general, most of the pipelines perform extremely poorly, and are not much better than a random guess with an accuracy of 0.5.

### 3.2 Classification between Target and Distractor stimuli

**Figure 5:**
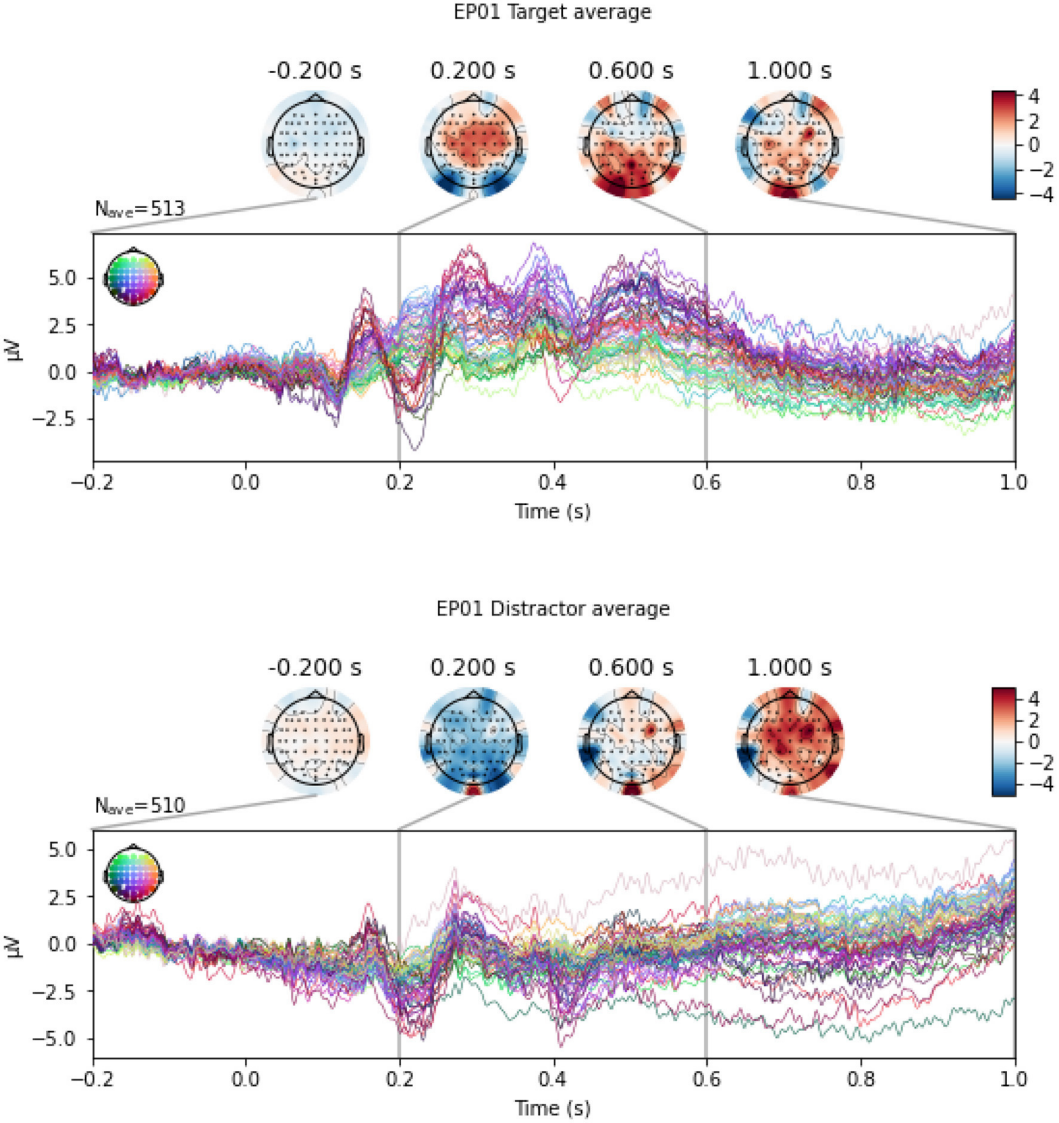
An example of EEG signals across 64 EEG electrodes for participant EP01, averaged across Target **(top panel)** and Distractor **(bottom panel)** stimulus.

In this section, we perform the classification between Target and Distractor stimuli, since P300 ERP is expected to be observed when subjects see a Target stimulus, and the amplitude of the P300 has shown to be proportional to the amount of attentional resources engaged in processing a given stimulus (Gray et al., 2004). Indeed, Mortier et al. (2023, Figure 2) shows that the evoked response for an example WithMe participant exhibits a clear positive voltage deflection around 300ms post-stimulus in the parietal-occipital electrodes. This can also be observed in Figure 5, which shows an example of EEG multivariate time series for a participant, for the different type of stimuli. Classification between Targets and Distractors investigates whether the brain responds differently to the two different stimulus types, which is thus informative of attention, but does not depend on the particular choice of the attention score.

Remember that the WithMe experiment is performed under four experimental conditions C1-C4, that indicate the presence of rhythmic or auditory support (Section 2.1). Whenever present, the rhythm and audio accompany the Targets only (and not the Distractors), and in order to ensure that the classification above is a differentiation between the Target and Distractor stimuli rather than a detection of rhythm and auditory support, we perform the classification separately under each condition. Note, moreover, that the subjects do not always correctly identify the Target or Distractor stimulus, so that their EEG signals do not necessarily exhibit a behavior that might be representative of the different type of stimuli. To avoid this issue, we limit the classification only to stimuli that were perfectly remembered or ignored (score = 0). Restricting the data in such a way results in somewhat different number of appropriate EEG epochs across participant, and we randomly select the minimum number of respectively 109, 84, 87, and 106 EEG epochs for conditions C1-C4.

**Figure 6:**
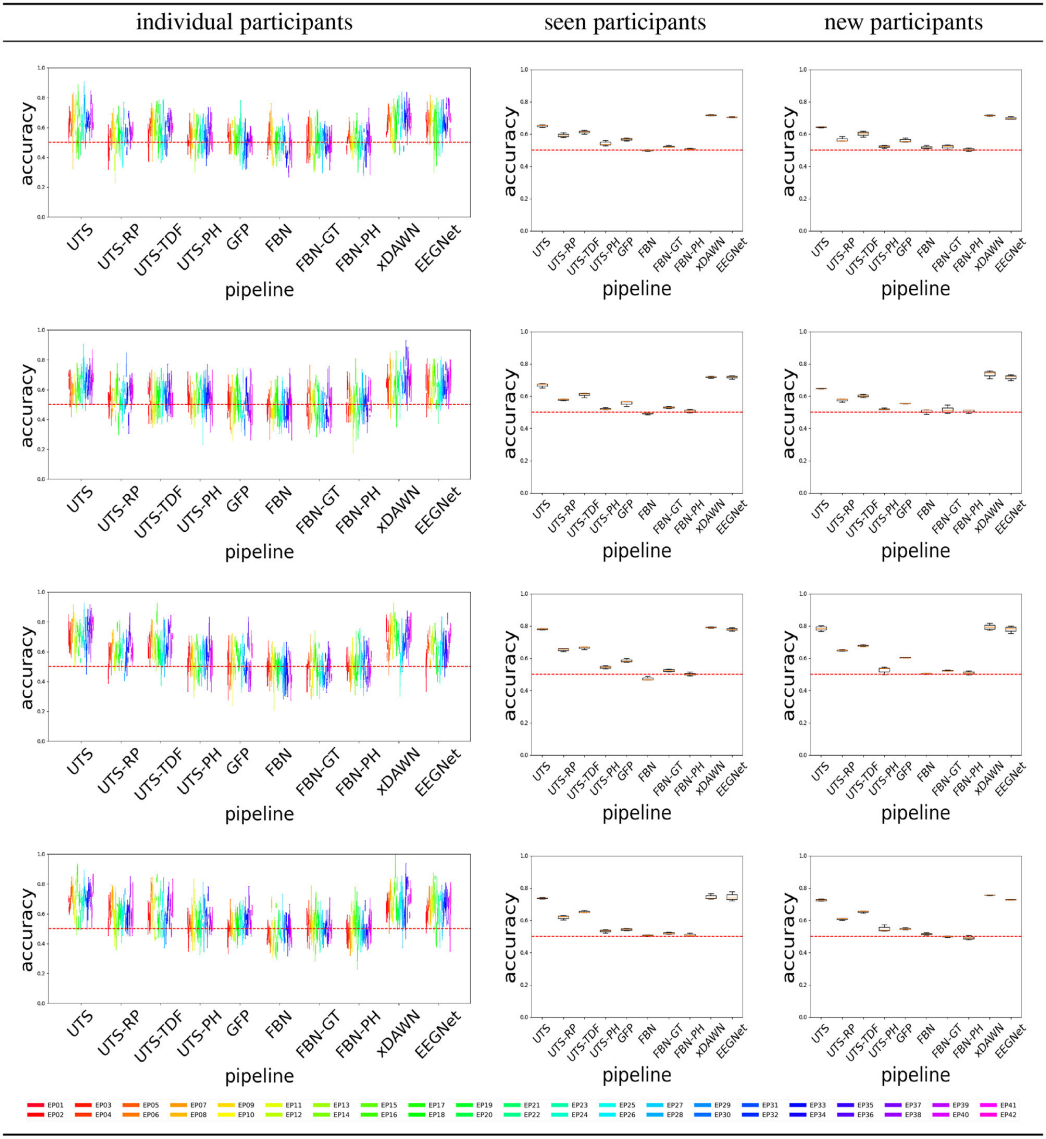
Target vs. Distractor classification accuracy across the pipelines, for conditions C1-C4 shown respectively from **(top to bottom row)**.

Figure 6 shows that a number of pipelines achieve a good classification accuracy of 75% or more. What is probably the most surprising is that the simplest pipeline UTS (Section 2.2.1), where the univariate time series across 64 EEG channels are simply concatenated into a large vector, obtains a very good performance, at times even outperforming the benchmark xDAWN-RG and deep learning EEGNet methods. Overall, in this case, the pipelines that extract and then concatenate the features from each of the univariate time series separately (UTS, UTS-RP, UTS-TDF, UTS-PH) outperform the pipelines that focus on the relationship between the time series across EEG channels (GFP, FBN, FBN-GT, and FBN-PH).

### 3.3 Classification between conditions C1, C2, C3, and C4

**Figure 7:**
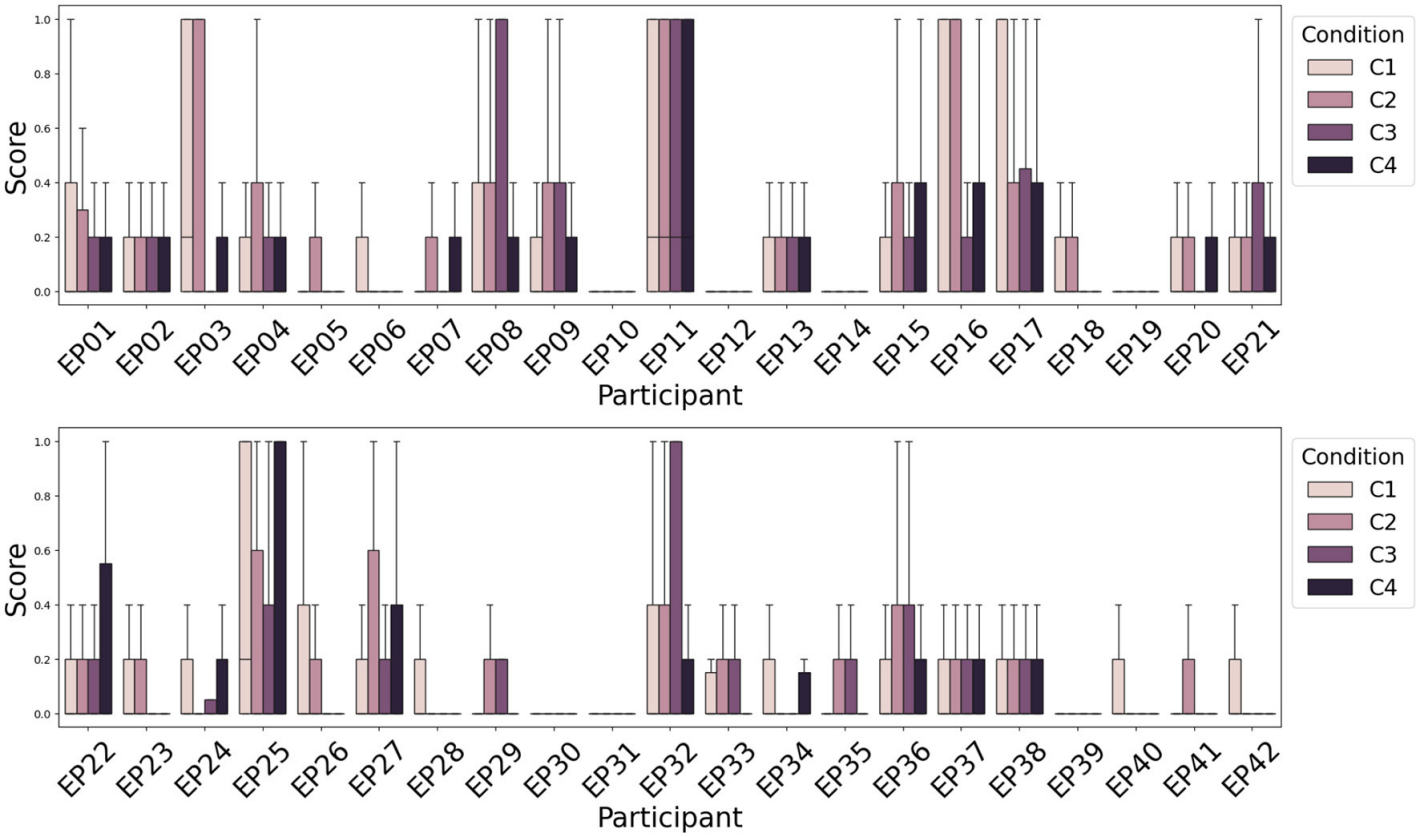
Average attention scores (lower is better) across participants and experimental conditions indicate that the influence of rhythmic and/or auditory support on the attention score is different across participants. Rhythm (C2) deteriorates the performance more often than improving attention. Auditory support (C3), however, improves the attention score for the majority of participants.

Next to attention recognition, one of the goals of the WithMe experiment (Section 2.1) is to investigate to what extent can rhythmic and/or auditory clues (Figure 1, right panel) improve attention. There is no significant difference among the distribution of attention scores, or scores averaged across participants, for the four experimental conditions C1-C4. This is because the perception of the auditory and/or rhythmic support turns out to be a very individual experience, improving the task performance for some, and causing distraction for other participants. We therefore rather visualize the attention score separately for each participant (Figure 7).

Figure 7 shows that for the majority of the participants, the presence of rhythm alone (C2) does not improve their attention score (Section 2.3) during the modified digit span task, although there are some differences across subjects. However, the presence of auditory support (C3) commonly helps to achieve a better score, that is rarely improved further with the additional rhythmic support (audio and rhythm together, C4). This is consistent with earlier findings on the WithMe data (De Winne et al., 2022), for three different scoring functions that look into the performances across complete sequences of digits shown on the screen (rather than for each of the 10 individual stimuli in a sequence).

**Figure 8:**
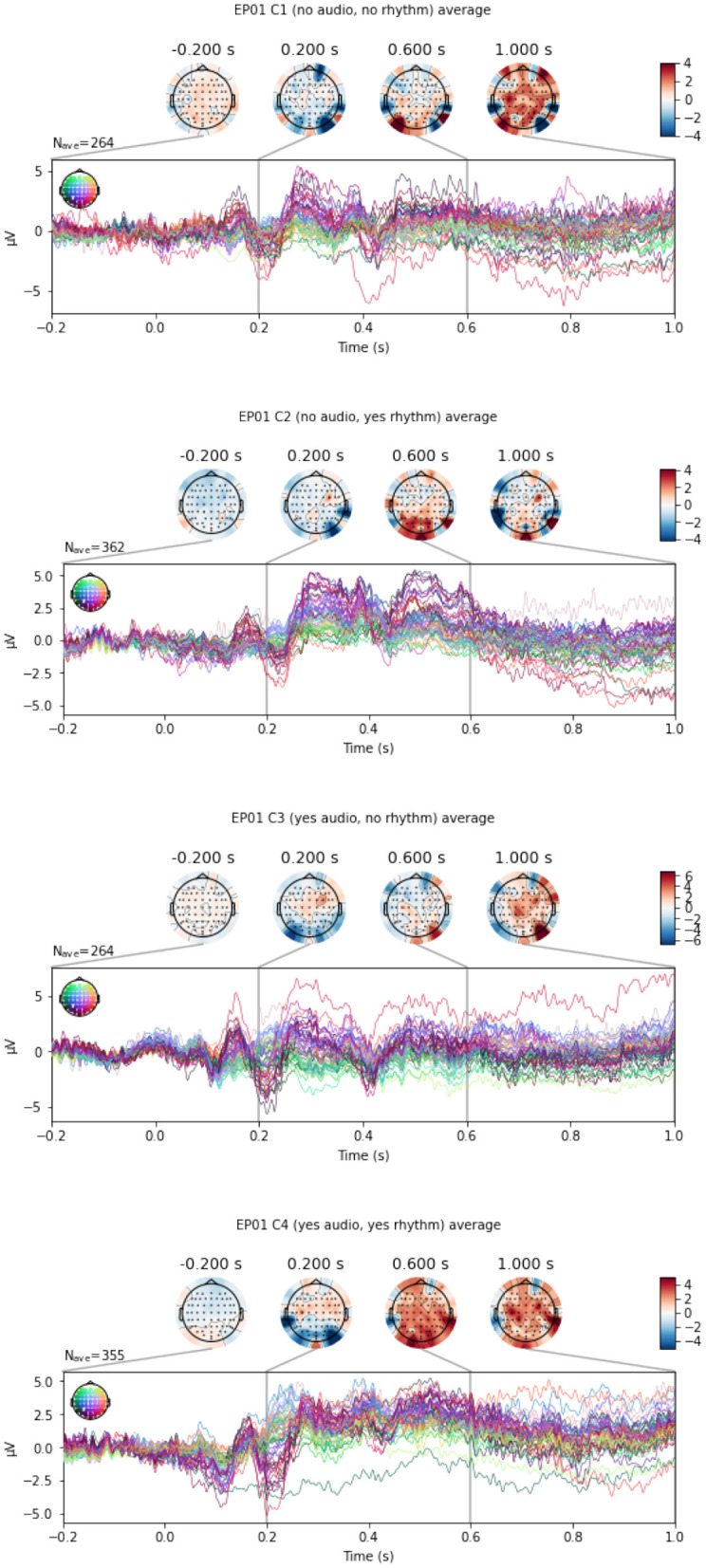
An example of EEG signals across 64 EEG electrodes for participant EP01, averaged across experimental conditions C1–C4, respectively from **(top to bottom panel)**.

In this section, we classify between the experimental conditions C1, C2, C3, and C4, to assess to what extent the EEG signals differ in the presence of rhythm and/or audio. An example of an EEG multivariate time series for a participant, across different conditions, is shown in Figure 8.

Since the auditory and/or rhytmic support only accompanies the Target, we perform the classification separately for Target and Distractor stimuli. We again also limit the data to EEG epochs with score = 0, resulting in somewhat different number of appropriate EEG epochs across participant; we randomly select the minimum number of respectively 137 and 224 EEG epochs for the two types of stimuli.

**Figure 9:**
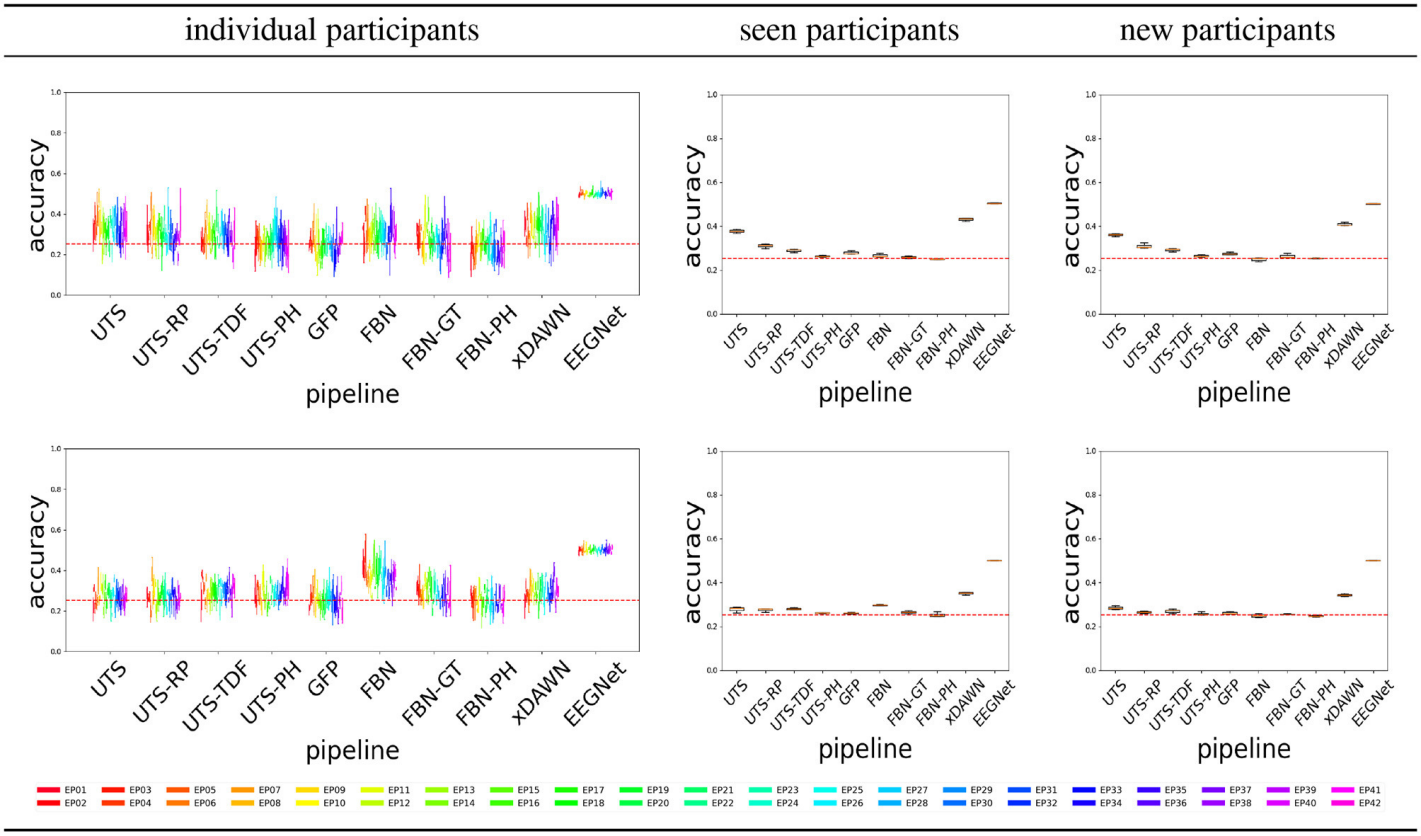
C1 vs. C2 vs. C3 vs. C4 classification accuracy across the pipelines, for the Target **(top row)** and Distractor **(bottom row)** stimuli.

The performance of the different EEG representations is rather poor for this task, never achieving the accuracy of the EEG-specific deep learning model EEGNet that learns the best features (Figure 9). The performance is even poorer for the Distractor stimuli, which comes as no surprise since there is no rhythm or audio that can change the EEG signal. However, a good performance of EEGNet in this case indicates that the support that is present during the Target stimuli in the sequence might help a person to correctly ignore the Distractor.

## 4 Discussion

### 4.1 Effectiveness of EEG representations in capturing attention

Attention can be captured with EEG, even for short visual stimulus. The performance for Target vs. Distractor classification we obtained is similar to the results from other comparable studies which obtain an accuracy between 56.5 and 84% (Mohamed et al., 2018, Table 3). For example, similar accuracy from EEG data of 76.82% is obtained when classifying attentive and inattentive students in Liu et al. (2013), and 84 and 81% for respectively the focused attention and working memory in Mohamed et al. (2018). Therefore, EEG-based BCI platforms have a good potential for utilization real-time classification and neurofeedback tasks, aiding in the diagnosis and training of individuals with attention deficits.

Overall, the representations of the EEG data that reflect the (features extracted from the) univariate time series perform better than the representations that focus on the relationship between these time series for different EEG channels. The former, including the recurrences in the time series, the common time-domain features, and the persistent homology (reflecting the local extrema) are thus all indicative of the use of attentional resources. The latter representations do at times obtain a superior performance on classification tasks for individual subjects, but they fail to obtain a good performance on previously unseen subjects. The communication between different regions in the brain is thus informative of processing visual stimuli (with rhythmic and/or auditory support), but is more sensitive to the cross-subject variability.

It is important to note that the insights might be strongly influenced by the particular problem (task at hand and the experimental set-up) and hyperparameters within the different pipelines. For instance, the WithMe experiment focuses on a novel digit-span paradigm with young students, and different EEG features might be most meaningful for other problems. The WithMe EEG time series reflect the brain signal during only 1.2 s of a simple visual task; more meaningful relationships between the different brain regions might be captured for longer resting-state time series. For example, Vandecappelle et al. (2021) show that the performance of the state-of-the-art models for classifying auditory attention drops significantly when shorter windows are used: the accuracy decreases by 17.6% going from 10 to 1 s. In the literature, most of the papers that focus on attention detection from EEG, aim to classify e.g., between long(er) reading or arithmetic task vs. resting or sleeping state. Moreover, functional brain networks are more common for diagnostic purposes from long resting-state fMRI data, as EEG has less discriminating power due to its limited spatial resolution (Yoo et al., 2014). Cross-task classification accuracies, where a classifier is trained and tested on EEG features from different tasks, has been found to be significantly lower (even than a random guess) compared to within-task condition (44.8 and 87.1%, respectively), since different tasks invoke highly dissimilar EEG patterns (Ke et al., 2014). In addition, relative contribution of different features of stress classification model has been shown to change with the infant's age (Lavanga et al., 2020). Our findings are also limited to our particular choice of the common Pearson correlation used to obtain the functional brain networks, whereas other measures of correlation might be more suitable in capturing attention from (raw) EEG. Furthermore, to address the issue of cross-subject variability, it might be a good idea to perform participant-based normalization. For example, Mohamed et al. (2018) use decibel conversion to normalize with respect to the baseline resting-state EEG data with open and closed eyes. However, the WithMe data does not include such resting state baselines, and we can thus recommend collecting such data during the experiment.

We should note that our goal is not to obtain the best possible performance. A better performance can likely be achieved by combining some complementary representations. For example, the correlation matrix and persistent homology (similar to our FBN and FBN-PH pipelines) combined has been shown to outperform the individual approaches for autism detection from fMRI data (Rathore et al., 2019). However, our goal is to gain insights in how powerful the different representations are, so that we evaluate the performance of each representation separately.

### 4.2 Good performance of raw time series

One of the most surprising insights from our experimental results is that the UTS pipeline (Section 2.2.1), which simply concatenates the univariate time series across EEG channels into a large vector, obtains a very good performance on some classification tasks, often outperforming the very complex EEG-specific models, although we have not observed its usage in the literature. It can therefore be recommended as a good starting baseline, and in case of good performance can avoid further intricate pre-processing and representation techniques (that require expertise in the application domain). This is in contradiction to the common understanding that extracting relevant features for the considered application is a crucial step in EEG processing (Xu et al., 2021). Moreover, we employ a simple SVM on the raw time series (in order to have a good indication of the discriminative power of the features), but better results can likely be achieved with more powerful learning models, such as multilayer perceptrons (MLPs) or other deep neural networks. A review of deep learning architectures in the analysis of EEG signals can be found in Craik et al. (2019).

### 4.3 Poor performance of persistent homology

Although topological data analysis, and its main tool, persistent homology, has found successful applications in neuroscience (see Sections 2.2.4, 2.2.8, Supplementary Material Section 1 for references and details), a closer look into the literature often points to a low effectiveness of these features, and a number of possible explanations.

The first TDA pipeline in this paper, UTS-PH (Section 2.2.4), calculates 0-dimensional PH on every univariate time series that reflects the EEG data for a single participant, stimulus and EEG channel. This captures the local extrema of the time series, but in case of the WithMe data these might contain a lot of noise, since e.g., a time series corresponding to a Target stimulus might contain the EEG information about the forthcoming Distractor(s). This might also explain why the UTS-TDF where the extrema are taken from the interesting range in the time series, or the UTS representation that considers the complete time series (and lets the classifier focus on the important information) outperform the UTS-PH pipeline. Persistent homology on univariate time series might be more likely effective in applications where the important difference between data classes lies in some extreme values of the signal, such as epileptic seizure, autism and arrhythmia detection from EEG or ECG (Wang et al., 2015, 2019; Majumder et al., 2020; Dindin et al., 2020).

The second TDA pipeline in this paper, FBN-PH (Section 2.2.8), calculates 0- and 1-dimensional PH on a functional brain 64 × 64 network reflecting the relationship between the time series across 64 EEG channels, for a single participant and stimulus. The poor performance in this paper is consistent with the experimental results in Gracia-Tabuenca et al. (2020, Figure 3), Rathore et al. (2019, Figure 4), and Guo et al. (2022), that point to limited or no success of PH features for brain functional networks. It is also important to note that PH on EEG-based functional brain networks has commonly been employed on correlations between the frequency domains, rather than the correlations between the time series themselves. In this paper, we focus on the latter approach, since frequency-domains for short time series provide poor frequency resolution.

Moreover, PH-based representations have previously been shown to be successful on much longer, resting-state fMRI data (more informative than EEG), and for diagnostic purposes (likely an easier task compared to detecting attention during a 1.2-second long visual stimulus). And even in such applications, success is not guaranteed: for instance, Gracia-Tabuenca et al. (2020) note only minor differences and small odd ratios between resting-state fMRI for diagnosing ADHD. There are only a few examples where PH has been used for neurotypical development, such as Gracia-Tabuenca et al. (2023) that however looks into the differences between PH on adolescent brain and random MRI networks. In addition, it is more common in the literature to show a statistically significant difference between the patients and the control group, rather than evaluating the accuracy of the more challenging classification or prediction tasks. The potential of PH to reveal the underlying processes from EEG during a short cognitive task might thus be limited.

We again note that the performance might be improved by combining the different representations. Indeed, a number of studies have suggested that persistent homology can extract features that are hardly noticed by other methods, so that their incorporation in deep learning models might yield better results. However, Rathore et al. (2019) provide a cautionary tale in this regard, as they show that the additional persistent homology features do not necessarily significantly improve the performance of deep learning models. The authors speculate that the poor performance might be attributed to the wide age group in their experiment, although persistent homology also underperforms on the WithMe data in this paper, where the participants are within a narrow 5-year range.

A survey of some promises and pitfalls of TDA for brain connectivity analysis is provided in Caputi et al. (2021). The field of applying topological data analysis, including persistent homology, to cognitive processes is an active area of research, and new studies and methods are continuously emerging. We encourage future research in this direction to help assess the effectiveness or added value of persistent homology in neuroscience applications.

### 4.4 Auditory and rhythmic support

Using only the WithMe behavioral data and participants' answers, De Winne et al. (2022) show the effect of auditory support, but no difference was observed between rhythmic and non-rhythmic sounds. These experiments focus on sequence-based scoring functions (that evaluate attention during a sequence of 10 digits), and we obtain similar findings for our stimulus-based attention score (Figure 7). To better understand the underlying processes of attention, as future work De Winne et al. (2022) suggest to also analyze the brain activation data such as EEG. Our results show that, although some pipelines can differentiate between the EEG data across experimental conditions C1-C4, there is little difference between EEG signals for the distractor stimuli that are not accompanied with rhythmic and/or auditory clues.

There is indeed prior evidence in the literature about the benefit of auditory support (Van der Burg et al., 2008), but the results about rhythm are conflicting. On the one hand, theories of dynamic attending and predictive coding suggest that rhythms generate expectations that open up slots for attending, facilitating memorization and recall of targets. On the other hand, the accuracy of task performance has been shown to not depend on the synchronization between the rhythm and target (Elbaz and Yeshurun, 2020; Huygelier et al., 2021). A possible explanation for the rhythm not providing additional support might lie in the model by Kahneman (1973), which suggests that attentional resources are drawn from a general, but limited pool of resources: memory requires the major part of available resources, so that not enough resources can be assigned to process the rhythmic support. This explanation is however less likely since healthy adults have on average a digit span between 5 and 9, so that memorizing 5 targets should not put too big of a demand on the available cognitive resources.

A more likely explanation can be found in a few methodological issues. Firstly, in order not to draw attention to the support and following the directed attention hypothesis, the conditions C1–C4 do not follow a block experimental design but the order is pseudo-randomized (so that even two auditory or rhythmic conditions would rarely appear consecutively), and participants were not made aware of the sound or rhythm (whereas some research suggests that conscious attention to both sensory modalities is essential for performance improvement, Van Ee et al., 2009). Indeed, even though the presence of rhythm did not improve attention in general, it did so for the participants who reported experiencing the rhythm as supportive (De Winne et al., 2022); however, it might be that these participants were more alert overall, what led to a better performance. Secondly, the priming of the rhythm with only five induction stimuli might have not been sufficient, and the induction with flashing empty circles might also be improved with moving stimulus such as a bouncing ball (De Winne et al., 2022).

We stress, however, a difference in the added value of auditory and/or rhythmic support across participants. This added value has been shown to be influenced by the audiovisual dominance, perceived audio/rhythm support and task difficulty, both in earlier studies as well as recently for the WithMe data (De Winne et al., 2022).^6^

### 4.5 Concluding remarks and future research

In this work, we examine the effectiveness of various EEG representations, derived from both univariate and multivariate time series, in relation to human attention. We observe that attention can indeed be captured with EEG, even for short visual stimulus; most of the common EEG representations demonstrate some utility for at least one of the three considered problems (attention score prediction, classification between Target and Distractor stimuli, classification between experimental conditions C1–C4 that indicate the presence of auditory and/or rhythmic support).

Notably, we observe that raw representations perform surprisingly well, while persistent homology features show very limited effectiveness. In Sections 4.2, 4.3, we provide a more detailed discussion of these findings; however, we encourage further application of these techniques across different tasks, to paint a more complete picture of their utility in EEG data analysis and neuroscience more broadly.

As anticipated, baseline benchmark models, such as EEGNet—a (black box) deep learning architecture designed to learn the best features for a given task— achieve the best performance in capturing attention. Although maximizing classification accuracy was not the primary objective of this study (instead, our focus was on evaluating the power of various EEG features), these models can be recommended when classification accuracy is of paramount importance.

Finally, the conflicting results regarding the influence of auditory and/or rhythmic stimuli on attention underscore the need for further research in this direction.

## Data Availability

The original contributions presented in the study are included in the article/supplementary material, further inquiries can be directed to the corresponding author.

## Footnotes

^1^ https://www.biosemi.com/Products_ActiveTwo.htm

^2^The manual inspection is the most common, “gold standard” procedure for bad (flat or excessively noisy channels) channel detection (Komosar et al., 2022). To maintain the same data dimensionality for every participant, bad channels are not removed, but rather interpolated. The interpolation is performed using the interpolate_bads() function of the MNE Python library, see: https://mne.tools/dev/documentation/implementation.html#channel-interpolation for details about the procedure.

^3^Figure 2 focuses on an example WithMe data point, but the pipelines can be applied to any data set of multivariate time series.

^4^These graph theory metrics have been calculated using the igraph Python library, see: https://igraph.org/r/doc for explicit formulas.

^5^After visual inspection of these graphs, we settled for the threshold value of 0.1, to draw a balance between graphs that are all very sparse or almost completely connected. Note that, as we discuss in Section 2.2.8, any method to choose the threshold is fairly *ad-hoc*.

^6^In De Winne et al. (2022), Kruskal-Wallis stastical test is based on the following independent variables: condition, sequence, order of the sequence of digits, musical education and practice, audiovisual dominance, gender, age, handedness, current emotional state, weekly physical activity, caffeine consumption, noise sensitivity, perceived tiredness, memory and task difficulty, and perceived sound/rhythm support. Female gender, pleasant emotion, and order of the sequence in the test also predict somewhat better performance (although interaction with the random participant variable demonstrates that some subjects show a learning, but others a fatigue effect), whereas the remainder of the personal factors are insignificant.
