# Supplementary material for: Who is WithMe? EEG features for attention in a visual task, with auditory and rhythmic support
Source: Front Neurosci. 2025 Jan 10;18:1434444. doi: 10.3389/fnins.2024.1434444 (PMC11758281; doi:10.3389/fnins.2024.1434444)
Supplement: Supplementary file 1 [file Presentation_1.pdf]

Supplementary Material

1 BACKGROUND ON TOPOLOGICAL DATA ANALYSIS

Topology has long been a central concept in neuroscience (Expert et al., 2019). In particular, persistent homology (PH), the main tool of topological data analysis, has found many applications. PH captures information about the k-dimensional cycles (connected components, loops, trapped volumes, etc.) in data (Zomorodian and Carlsson, 2005; Edelsbrunner and Harer, 2008; Adams et al., 2017). The data is commonly a point cloud $X \subset \mathbb{R}^n$, but it can also be a graph, image, or any real function. To calculate PH, we first construct a so-called filtration $\{K_r\}_{r \in \mathbb{R}}$ which approximates X at different scales $r \in \mathbb{R}$. For a point cloud, a standard choice for K_r is the common Vietoris-Rips simplicial complex, which approximates the structure of the point cloud at resolution r by constructing an edge between two points whenever they are within distance r. For a homological dimension k, PH registers the birth and death time r of every k-dimensional cycle within the filtration, which might reflect some additional geometric information about the cycles, such as their size or position. PH is commonly described with a scatter plot of birth and death coordinates, referred to as persistence diagram (PD). As scatter plots, the complicated structure of PDs makes them inconvenient for statistical inference, so that they are commonly vectorized into so-called persistence images (Adams et al., 2017) or landscapes (Bubenik, 2015). The pipeline to extract PH features is visualized in Figure S1.

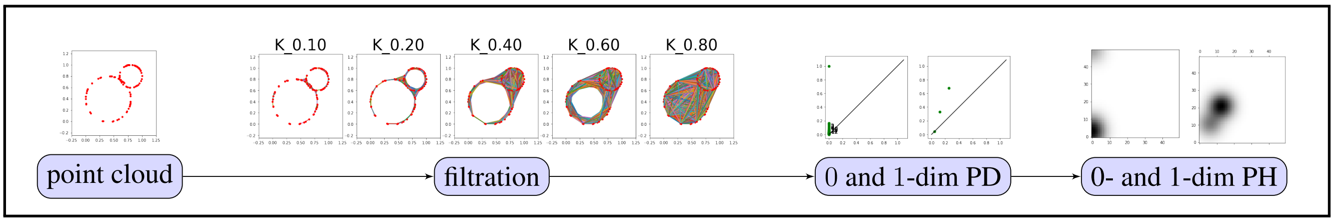

Figure S1. Persistent homology features. For the given point cloud in $\mathbb{R}^2$, PH is calculated on the common Vietoris-Rips filtration that approximates the point cloud at different scales. 0-dimensional PD has one persistent cycle, reflecting the single connected component, and a number of short cycles that correspond to the individual point-cloud points that are connected to other ones early in the filtration. 1-dimensional PD summarizes the two holes, whose birth and death values respectively reflect the sparsity along the hole and the size of the hole, as these are the scales $r \in \mathbb{R}$ at which the hole appears and when it is filled in within the filtration. PDs can then be represented with a vector signature such as by persistence images (PIs), that can directly be used in statistical learning frameworks.

One reason behind the application of PH to neuroscience is that a significant portion of neuroscience research involves studying functions or graphs, that are particularly amenable to topological tools (Curto, 2017). The functions can, for instance, correspond to the functional magnetic resonance imaging (fMRI) time series, and graph vertices can correspond to neurons, cell types, fMRI voxels, with the graph edges reflecting their relationship (e.g., synaptic connections between neurons). A survey of applications of topology in neuroscience can be found in (Curto, 2017). Some examples of application of PH in neuroscience are analysis of: spatial structure of afferent neuron terminals in crickets (Brown and Gedeon, 2012), correlations of neurons in the hippocampus (Giusti et al., 2015), neuron activity (firing event time series, so-called spike trains) (Spreemann et al., 2015), branching neuronal trees (Kanari et al., 2018), brain artery trees (Bendich et al., 2016; Biscio and Møller, 2019), morphological brain networks in deaf

adults (Kim et al., 2014), fluorodeoxyglucose-positron emission tomography (FDG-PET) based brain networks in children with attention deficit hyperactivity disorder (ADHD) and autism (Lee et al., 2011, 2012), functional magnetic resonance imaging (fMRI) volumes (Rieck et al., 2020), fMRI brain networks (Stolz, 2014; Petri et al., 2014; Ellis and Klein, 2014; Stolz et al., 2018) (respectively during learning, drug-induced states, in patients with ADHD or schizophrenia), or structural covariance MRI (scMRI) or fMRI networks of persons with autism (Palande et al., 2017; Rathore et al., 2019).

There are a few different ways that PH can be calculated from EEG data (or any dataset of multivariate time series). For instance, one can compute PH on a point cloud, a geometric object obtained from a time series via the so-called Takens's time delay embedding, which is circular if the time series is periodic, under certain conditions (Perea and Harer, 2015). Since 1-dimensional PH precisely describes the loops in the data, it provides insights into periodic and repetitive patterns in the time series, and promises to be beneficial in particular to distinguish periodic and chaotic time series (Seversky et al., 2016). In such applications, time series belonging to the same class do not have to have similar waveform and similar range, so that this PH-based model might be more meaningful than the typical approaches for time series classification (e.g., temporal or frequency features, or CNNs that learn the best features for the given task). It is possible to choose a summary of PH that is shape agnostic and resistant to dampening (Perea et al., 2015), what might be useful in dealing with cross-subject variability. This pipeline has been employed on EEG data for epileptic seizure (Ghadyali, 2017) or cognitive fatigue detection (Das et al., 2020), classification between eyes-open and eyes-closed signals (Bischof and Bunch, 2021), or between left and right-hand motor intentions for brain-computer interface (BCI) applications (Altındiş et al., 2021; Altindis et al., 2018). A recent review of TDA applications to EEG analysis can be found in (Xu et al., 2021).

In this paper, we apply PH both on univariate time series for a single EEG channel (i.e., functions, `UTS-PH`, Section 2.2.4 and on the networks resulting from the EEG multivariate time (i.e., graphs, `FBN-PH`, Section 2.2.8 . In the remainder of this section, we discuss our choice of filtration and persistence signature, the input and output of PH.

1.1 Filtrations

Next to the common Vietoris-Rips filtration, there is a number of different ways one can build a filtration, the input for PH. PD always registers the number of k-dimensional cycles (topological information), but the choice of filtration has crucial importance for the interpretation of birth and death values (additional geometric information), and for the type of data transformations that the resulting PH is invariant to. For example, the birth and death values with respect to the Vietoris-Rips filtration reflect respectively the sparsity of the neighbouring point cloud points and their radius or size (Figure S1), so that they are invariant under rotation or translation, but not under rescaling or outliers. In this section, we focus our attention on the two filtrations that we will use in this work.

1.1.1 Lower-star filtration

Let $f : \mathbb{R} \to \mathbb{R}$ be a function. The lower-star filtration $\{K_r\}_{r\in\mathbb{R}}$ considers the sublevel sets

$$K_r = \{x \in \mathbb{R} \mid f(x) \leq r\}.$$

Every sublevel set K_r is either empty or a union of intervals, so the only non-trivial topological information they carry is their 0-dimensional homology, i.e., the number of connected components (Chazal and Michel, 2021). Since PH tracks how these connected components evolve within the filtration, it will capture the

information about the local minima and maxima of f (Figure S2). Note that PH does not capture the width of the minima and maxima peaks, so that it is invariant to expansion and contraction in the x-axis direction.

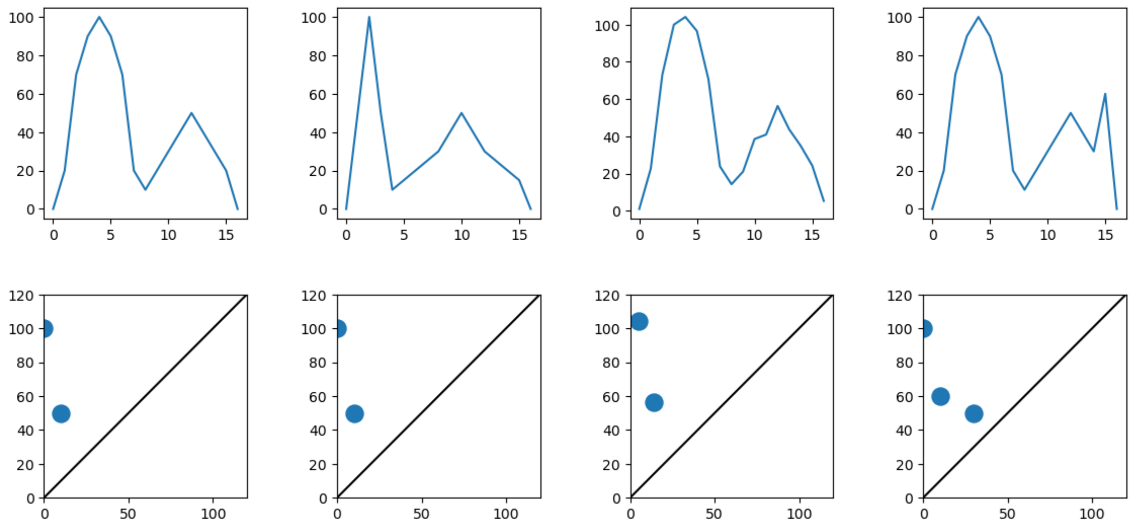

Figure S2. Persistent homology of a function with respect to the lower-star filtration captures the information about the local maxima and minima of the function, and is invariant under expansion and contraction in the time axis direction. The figure shows four example functions $f : \mathbb{R} \to \mathbb{R}$ (top panel) and their respective 0-dimensional PDs (bottom panel). For the first example f_1, $\text{PD}_1 = \{[0, 100], [10, 50]\}$, reflecting the minimum values 0 and 10, and maximum values 100 and 50. Since the second function f_2 has the same extrema, $\text{PD}_2 = \text{PD}_1$, despite the width of the extrema being different. Function f_3 is a noisy version of f_1, resulting in a slightly different persistence intervals $\text{PD}_3 = \{[5.27, 104.17], [14.24, 56.32]\}$. Function f_4 has additional extrema, so that the PD sees additional persistence interval, $\text{PD}_4 = \{[0, 100], [10, 60], [30, 50]\}$.

1.1.2 Rank filtration

Let $G = (V, E)$ be a weighted graph or a network. In general, an algebraic representation of a weighted graph $G = (V, E)$ is a connectivity matrix $D = [d_{ij}]$, where d_{ij} is the weight of an edge $e_{ij} \in E$ between vertices $v_i \in V$ and $v_j \in V$, reflecting the relationship between them, which is typically some measure of distance, dissimilarity or inverse correlation. It is possible to build the standard Vietoris-Rips filtration of a graph, since it never relies on the point cloud coordinates, but rather only on the pairwise distances d_{ij} between the point cloud points (or graph vertices or nodes).

In applications, however, it is often the case that these graph distances are measured in somewhat arbitrary units, where one can usually assume a larger weight means a stronger interaction, but the precise values of the weights may not be meaningful (Curto, 2017). This is particularly true for neuroscience applications, where correlations are believed to be given by an unknown monotonic function on an underlying distance in the relevant stimulus space, i.e., distances are only known up to rescaling by an unknown monotonic function (Giusti et al., 2015; Bubenik et al., 2020).

In such scenarios, it is useful to consider the rank filtration, which corresponds to the Vietoris-Rips filtration, but after the scalar values in the distance matrix are replaced by their ordinal numbers or ranks. In other words, the rank filtration is a nested family of graphs (clique complexes of G), where each subsequent graph includes an additional edge $(i, j) \in E$ corresponding to the next-lowest distance d_{ij}. PH with

respect to the weight rank filtration is then a topological invariant, as it remains unchanged under nonlinear transformations of the weights, provided that the ordering of weights is preserved (Figure S3).

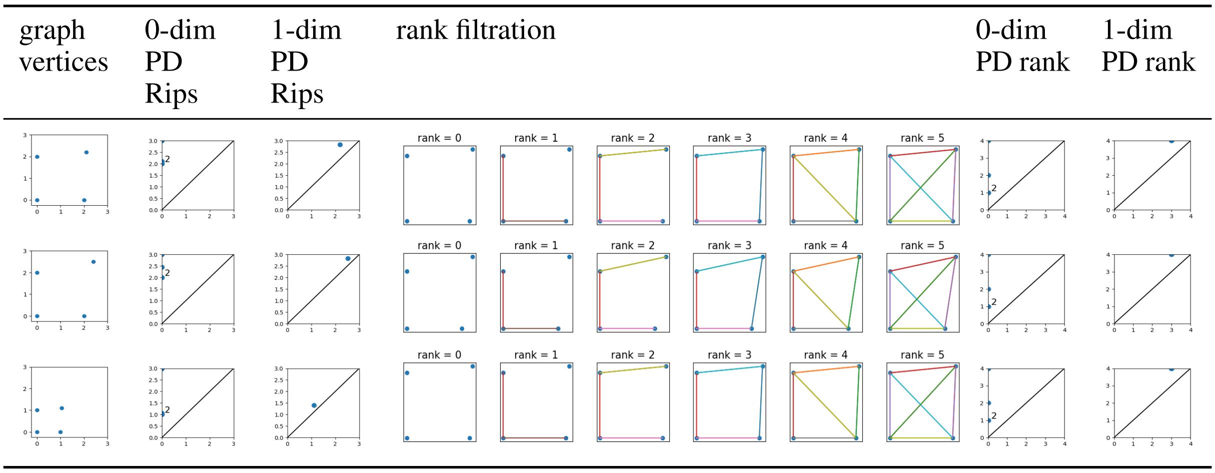

Figure S3. Persistent homology of a graph with respect to the rank filtration captures the connectivity and holes, invariant under rescaling of distances by a monotonic function. The 0- and 1-dimensional PDs with respect to either the standard Vietoris-Rips or the rank filtration reveal respectively the four connected components and one hole. However, the persistence intervals (i.e., their birth and death values) with respect to the standard Vietoris-Rips filtration differ for the three example graphs, since they rely on the actual distances between graph nodes. The rank filtration instead only considers the order of distances, so that the PDs are the same for the three graph examples.

1.2 Persistence signatures

The space of persistence diagrams (PDs), the standard signatures for PH, is hard to work with, e.g., sets of PDs do not even need to have a unique mean (Bubenik, 2015). In applications, one therefore typically relies on some vectorized summary of PDs. In this paper, we summarize PH with persistence images (PIs) (Adams et al., 2017), which is one of the most common choices in the literature. To calculate a PI, a grid is superimposed over a PD, and the volume below the weighted sum of Gaussian probability density functions is calculated on each grid cell. This can be seen as a more refined approach to counting the cycles within each of the cells of the grid.

2 DIFFERENT REPRESENTATIONS OF AN EXAMPLE MULTIVARIATE TIME SERIES

In this appendix, we consider two toy examples of 5×100 multivariate time series. For the first example, the first univariate time series is a cosine signal with a few minima and maxima. The remaining univariate time series in this example are obtained by applying respectively the following transformations to the starting signal: a shift in the y-axis direction, “stretching" a part of the time series in the x-axis direction, and adding two different levels of noise. The second multivariate time series example consists of the same first univariate time series, with the last one being replaced with a completely noisy signal. Figure S4 visualizes the different representations of the multivariate time series considered in this work (Section 2.2) , and gives some insights about the noise robustness of different features.

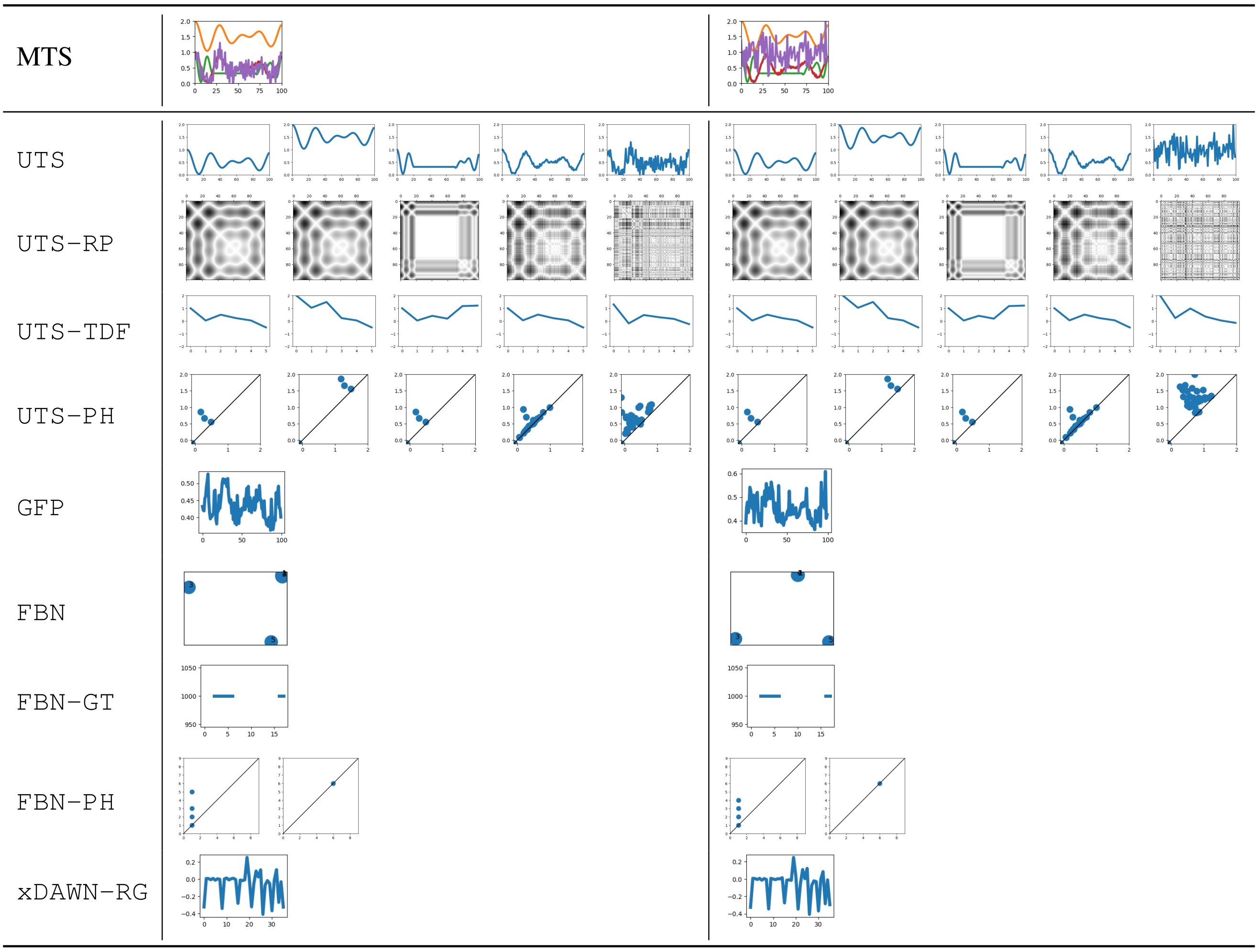

Figure S4. Different representations of two example multivariate time series (MTS).

REFERENCES

Adams, H., Emerson, T., Kirby, M., Neville, R., Peterson, C., Shipman, P., et al. (2017). Persistence images: A stable vector representation of persistent homology. *The Journal of Machine Learning Research* 18, 218–252

Altindis, F., Yilmaz, B., Borisenok, S., and Icoz, K. (2018). Use of topological data analysis in motor intention based brain-computer interfaces. In *2018 26th European Signal Processing Conference (EUSIPCO)* (IEEE), 1695–1699

Altındiş, F., Yılmaz, B., Borisenok, S., and İçöz, K. (2021). Parameter investigation of topological data analysis for EEG signals. *Biomedical Signal Processing and Control* 63, 102196

Bendich, P., Marron, J. S., Miller, E., Pieloch, A., and Skwerer, S. (2016). Persistent homology analysis of brain artery trees. *Annals of Applied Statistics* 10, 198

Bischof, B. and Bunch, E. (2021). Geometric feature performance under downsampling for EEG classification tasks. *arXiv preprint arXiv:2102.07669*

Biscio, C. A. and Møller, J. (2019). The accumulated persistence function, a new useful functional summary statistic for topological data analysis, with a view to brain artery trees and spatial point process applications. *Journal of Computational and Graphical Statistics* , 1–21

Brown, J. and Gedeon, T. (2012). Structure of the afferent terminals in terminal ganglion of a cricket and persistent homology. *PLOS ONE* 7, e37278

Bubenik, P. (2015). Statistical topological data analysis using persistence landscapes. *The Journal of Machine Learning Research* 16, 77–102

Bubenik, P., Hull, M., Patel, D., and Whittle, B. (2020). Persistent homology detects curvature. *Inverse Problems* 36, 025008

Chazal, F. and Michel, B. (2021). An introduction to topological data analysis: fundamental and practical aspects for data scientists. *Frontiers in Artificial Intelligence* 4, 108

Curto, C. (2017). What can topology tell us about the neural code? *Bulletin of the American Mathematical Society* 54, 63–78

Das, A. K., Kumar, K., Gavas, R. D., Jaiswal, D., Chatterjee, D., Ramakrishnan, R. K., et al. (2020). Cognitive fatigue detection from EEG signals using topological signal processing. In *2020 28th European Signal Processing Conference (EUSIPCO)* (IEEE), 1313–1317

Edelsbrunner, H. and Harer, J. (2008). Persistent homology-a survey. *Contemporary Mathematics* 453, 257–282

Ellis, S. P. and Klein, A. (2014). Describing high-order statistical dependence using “concurrence topology", with application to functional MRI brain data. *Homology, Homotopy and Applications* 16, 245–264

[Dataset] Expert, P., Lord, L.-D., Kringelbach, M. L., and Petri, G. (2019). Topological neuroscience

Ghadyali, H. (2017). *Applications of topological data analysis and sliding window embeddings for learning on novel features of time-varying dynamical systems*. Ph.D. thesis, Duke University

Giusti, C., Pastalkova, E., Curto, C., and Itskov, V. (2015). Clique topology reveals intrinsic geometric structure in neural correlations. *Proceedings of the National Academy of Sciences* 112, 13455–13460

Kanari, L., Dłotko, P., Scolamiero, M., Levi, R., Shillcock, J., Hess, K., et al. (2018). A topological representation of branching neuronal morphologies. *Neuroinformatics* 16, 3–13

Kim, E., Kang, H., Lee, H., Lee, H.-J., Suh, M.-W., Song, J.-J., et al. (2014). Morphological brain network assessed using graph theory and network filtration in deaf adults. *Hearing Research* 315, 88–98

Lee, H., Chung, M. K., Kang, H., Kim, B.-N., and Lee, D. S. (2011). Discriminative persistent homology of brain networks. In *2011 IEEE International Symposium on Biomedical Imaging: From nano to macro* (IEEE), 841–844

Lee, H., Kang, H., Chung, M. K., Kim, B.-N., and Lee, D. S. (2012). Weighted functional brain network modeling via network filtration. In *NIPS Workshop on Algebraic Topology and Machine Learning* (Citeseer), vol. 3

Palande, S., Jose, V., Zielinski, B., Anderson, J., Fletcher, P. T., and Wang, B. (2017). Revisiting abnormalities in brain network architecture underlying autism using topology-inspired statistical inference. In *International Workshop on Connectomics in Neuroimaging* (Springer), 98–107

Perea, J. A., Deckard, A., Haase, S. B., and Harer, J. (2015). SW1PerS: Sliding windows and 1-persistence scoring; discovering periodicity in gene expression time series data. *BMC Bioinformatics* 16, 257

Perea, J. A. and Harer, J. (2015). Sliding windows and persistence: An application of topological methods to signal analysis. *Foundations of Computational Mathematics* 15, 799–838

Petri, G., Expert, P., Turkheimer, F., Carhart-Harris, R., Nutt, D., Hellyer, P. J., et al. (2014). Homological scaffolds of brain functional networks. *Journal of The Royal Society Interface* 11, 20140873

Rathore, A., Palande, S., Anderson, J. S., Zielinski, B. A., Fletcher, P. T., and Wang, B. (2019). Autism classification using topological features and deep learning: A cautionary tale. In *International Conference on Medical Image Computing and Computer-Assisted Intervention* (Springer), 736–744

Rieck, B., Yates, T., Bock, C., Borgwardt, K., Wolf, G., Turk-Browne, N., et al. (2020). Uncovering the topology of time-varying fMRI data using cubical persistence. *Advances in Neural Information Processing Systems* 33, 6900–6912

Seversky, L. M., Davis, S., and Berger, M. (2016). On time-series topological data analysis: New data and opportunities. In *Proceedings of the IEEE Conference on Computer Vision and Pattern Recognition Workshops*. 59–67

Spreemann, G., Dunn, B., Botnan, M. B., and Baas, N. A. (2015). Using persistent homology to reveal hidden information in neural data. *arXiv preprint arXiv:1510.06629*

Stolz, B. (2014). Computational topology in neuroscience. *Master's thesis (University of Oxford, 2014)*

Stolz, B. J., Emerson, T., Nahkuri, S., Porter, M. A., and Harrington, H. A. (2018). Topological data analysis of task-based fMRI data from experiments on schizophrenia. *arXiv preprint arXiv:1809.08504*

Xu, X., Drougard, N., and Roy, R. N. (2021). Topological data analysis as a new tool for EEG processing. *Frontiers in Neuroscience* 15, 761703

Zomorodian, A. and Carlsson, G. (2005). Computing persistent homology. *Discrete & Computational Geometry* 33, 249–274
